# An ALE meta-analytical review of the neural correlates of abstract and concrete words

**DOI:** 10.1038/s41598-021-94506-9

**Published:** 2021-08-03

**Authors:** Madalina Bucur, Costanza Papagno

**Affiliations:** 1 CeRiN (Center for Cognitive Neurorehabilitation), Center for Mind/Brain Sciences (CIMeC), University of Trento, Via Matteo del Ben 5/b, 38068 Rovereto, TN Italy; 2 Department of Psychology, University of Milano-Bicocca, Milan, Italy

**Keywords:** Cognitive neuroscience, Neuroscience

## Abstract

Several clinical studies have reported a double dissociation between abstract and concrete concepts, suggesting that they are processed by at least partly different networks in the brain. However, neuroimaging data seem not in line with neuropsychological reports. Using the ALE method, we run a meta-analysis on 32 brain-activation imaging studies that considered only nouns and verbs. Five clusters were associated with concrete words, four clusters with abstract words. When only nouns were selected three left activation clusters were found to be associated with concrete stimuli and only one with abstract nouns (left IFG). These results confirm that concrete and abstract words processing involves at least partially segregated brain areas, the IFG being relevant for abstract nouns and verbs while more posterior temporoparietal-occipital regions seem to be crucial for processing concrete words, in contrast with the neuropsychological literature that suggests a temporal anterior involvement for concrete words. We investigated the possible reasons that produce different outcomes in neuroimaging and clinical studies.

## Introduction

An advantage for concrete words as compared to abstract words has been demonstrated in a series of psycholinguistic studies. Neurologically unimpaired participants perform better on concrete than abstract words in free recall, cued recall, paired-associate learning and recognition; their reaction times in visual lexical decision are shorter with concrete than abstract words^1^. This effect is known as “concreteness effect”, and it increases in aphasic patients. This is especially evident in non-fluent aphasia, for example in patients with agrammatism^2^, where it has been found in spontaneous speech^3^, reading^4^, writing^5^, repetition^6^, naming^7^, and comprehension^8^. Several theories^9–12^ have been proposed to explain this advantage of concrete words but they share a common feature, namely a quantitative distinction between concrete and abstract concepts, with concrete items more strongly represented than abstract ones, either because they benefit from a verbal and visuo-perceptual representation^10^ or thanks to a larger contextual support^12^ or a larger number of semantic features^9,11^. For instance, the Dual Coding Theory^10^ postulates that concrete concepts are supported by both perceptual and verbal representations while abstract words are based exclusively on linguistic information. From the Dual Coding Theory perspective, the advantage of concrete compared to abstract concepts is attributed to the additional contribution of the sensory-motor systems triggered by imagery-based richer representations, presumably involving both hemispheres (not only the left hemisphere) and to a greater number of units activated in the semantic system for concrete words^10^. The hub-and-spokes model assumes that words are processed in a neural network containing one or more amodal hubs, sensorimotor modality-specific regions, and connections between them (cross-modal conjunctive representations^13,14^. Initially, it was hypothesized that the anterior temporal lobes (ATLs) were the main hub, but, later on, other potential high-order and low-order hubs have been introduced (e.g., left posterior cingulate cortex, dorsomedial pre-frontal cortex, inferior frontal gyrus, inferior parietal cortex, precuneus)^15,16^. From the Dual Hub Theory^17,18^ perspective the ATL processes taxonomic knowledge (shared features, e.g., dog → wolf) while the temporo-parietal areas, including the posterior middle temporal gyrus (pMTG), are involved in thematic knowledge (contiguity relations based on co-occurrence in events or scenarios, e.g., dog—leash).

However, these theories cannot explain the reversal of concreteness effect that has been documented in a number of brain-damaged patients, both single cases^19–29^, and group studies^30–34^, who consistently show better performance on abstract as compared to concrete words.

To account for the reversed concreteness effect, it has been proposed that abstract and concrete concepts are distinguished by the manner in which they are acquired, and by the relative weight of sensory-perceptual features in their representation^20^. An alternative explanation by Crutch and Warrington^35^, points to a fundamental difference in the architecture of concrete and abstract word representations: the primary organization of concrete concepts is categorical, whereas abstract concepts are predominantly represented by association to other items. In this framework, a reversed concreteness effect might result from selective damage to categorical information (which would selectively affect conceptual representations of concrete words).

The selective impairment of concrete and abstract concepts suggests different anatomical correlates. In aphasic patients, an increase of the concreteness effect has been associated to vascular damage in the territory of the left middle cerebral artery, involving the prefrontal cortex. Cases of reversed concreteness effect, in contrast, are associated to herpes simplex encephalitis^26,29^ and semantic dementia both in single cases^20–22,25^, and group studies^30–32,34^, that typically affect anterior temporal regions. These results have been confirmed in patients after left temporal pole resection^33^ and during direct electrical stimulation in awake surgery^36^. All these data seem to suggest a role of the left prefrontal cortex and the anterior temporal lobe, in processing abstract and concrete concepts, respectively. Notably, with the exception of Yi et al.’s^34^ and Bonner et al.’s^30^ studies, the reversal of concreteness effect has been found for nouns but not for verbs.

Neuroimaging data, however, do not totally match clinical evidence. Indeed, while the role of the left inferior frontal gyrus (IFG) for abstract words is confirmed, a previous meta-analysis^37^, based on 19 fMRI and PET studies, also showed an activation of the middle temporal gyrus (MTG), and, crucially, concrete concepts compared to abstract ones seem to activate the left posterior cingulate, precuneus, fusiform gyrus, parahippocampal cortex, therefore, posterior regions. However, Wang et al.^37^ took into consideration not only nouns and verbs, but also sentences and fixed expressions, such as idioms. Thus, we hypothesized that this incongruence between clinical and neuroimaging studies could partly depend on the use of very different type of stimuli.

The present systematic review and meta-analysis aimed at addressing which regions are consistently activated across experiments that require participants to process abstract and concrete words, trying to adopt more stringent criteria considering type of stimuli and modality of presentation (visual or auditory). The rationale of these sub-analyses is based on the fMRI literature suggesting that stimulus type, presentation modality, but also tasks could impact on the pattern of activation^38,39^. Accordingly, we did not include studies using complex stimuli as sentences, or short stories since these publications might tap on different cognitive processes including for example attention and working memory.

Consequently, our study differs from previous meta-analyses^37,40^ in two aspects:

1. We exclusively selected papers that used only words stimuli and presented specific contrasts (concrete > abstract and abstract > concrete stimuli).
2. We used a different method, choosing the more popular Activation Likelihood Estimation^41–43^ (ALE) as compared to the multilevel kernel density analysis (MKDA)^44^ applied by Wang et al.^37^. MKDA and ALE produce similar results, both using the location (xyz-coordinates) of local maxima reported by the individual studies, but MKDA uses a spherical kernel whose radius is determined by the analyst^45^ while ALE applies a Gaussian kernel whose FWHM is empirically determined. Moreover, our analyses are conducted on the last version of the GingerAle software, which managed to rectify some of the previous limitations of this instrument, e.g., the frequently used FDR correction is no longer supported^43^ and proposes new best-practice ALE recommendations like the cluster-level family-wise error (FWE) corrected threshold of *p* < 0.05^46^.

Finally, this is also an update of the previous reviews, including publications from the last 10 years.

## Materials and methods

The present systematic review was conducted under the Preferred Reporting Items for Systematic Reviews and Meta-Analyses (PRISMA) guidelines^47^.

### Studies selection

Our meta-analysis is based on 32 neuroimaging studies exploring the neural basis of concrete and abstract words processing, using either PET or fMRI on adult participants, published between January 1996 and February 2021. Studies were selected using four electronic databases: MEDLINE (accessed by PubMed, https://www.ncbi.nlm.nih.gov/pubmed), PsycARTICLES (via EBSCOHost, https://search.ebscohost.com), PsycINFO (via EBSCOHost) and Web of Science (https://webofknowledge.com/). The search terms used were: (1) “semantic decision”, “semantic judgment”; “abstract words”, “concrete words “, “abstract concepts”, “concrete concepts”, “lexical decision” AND (2) “imaging”, “MRI”, “PET”. Additional sources such as reference lists of included studies and relevant systematic reviews were also checked.

Titles, abstracts, and full-text articles were screened and evaluated for eligibility based on the following criteria:

Inclusion criteria:

- Imaging technique: PET or fMRI,
- Reported stereotaxic coordinates (in the MNI or Talairach atlases),
- Whole-brain voxel-based data analyses,
- More than 5 participants in each study,
- Sample population of healthy, adult participants,
- Reported concrete > abstract or abstract > concrete contrast,
- Word stimuli,
- Published in English,

Exclusion criteria:

- Region-of-interest analyses,
- Multiple single-case analyses,
- Sample population of minors,
- Sample population of neurological, brain-damaged, cognitively impaired or psychiatric patients,
- Only concrete > baseline or abstract > baseline contrasts,
- Articles from the gray literature (i.e., literature that is not formally published in sources such as books or journal articles, e.g. unpublished Ph.D. thesis),
- Presentations from international meetings with no specific data provided, perspective and opinion publications, case reports, series of cases, previous reviews or meta-analyses,
- Studies not published in, or translated into English,
- Phrases or sentences stimuli,
- Studies without adequate information (e.g., stereotaxic coordinates) to analyze the concrete vs. abstract contrasts and no reply from the authors after asking for the missing data.

We used a general and broad initial search. We looked for publications that reported word stimuli and concrete > abstract or abstract > concrete MRI and PET contrasts. In a second time, we distinguished the included papers taking into consideration the type of knowledge, type of task, type of stimuli, and type of investigation method. These classifications led to exploratory sub-analyses, with the purpose of controlling as much as possible confounding factors. As previously specified, we looked for publications that reported concrete > abstract words or abstract > concrete words contrast, without analyzing the exact strategies that the authors applied to divide word stimuli into the two categories. Often, the abstractness/concreteness constructs are operationalized in the papers based on two rating methods: (1) asking participants to classify a word as concrete taking into consideration the degree to which it refers to a tangible entity in the world (it has clear references to material objects); (2) or by evaluating its imageability, i.e., the ease with which the word elicits a mental image. Generally speaking, words referring to something that exists in reality, and one can have an immediate experience of it through the senses are considered concrete (e.g., animals, tools); while words whose meaning cannot be experienced directly but can be defined by other words, internal sensory experience, and linguistic information, are classified as abstract (e.g., emotions, morality, social interaction, time).

After removing duplicates, research papers which did not satisfy the above criteria were excluded. For example, several studies focused on sentences or phrases^48,49^; or reported only words > baseline contrasts^50^. The more conservative concrete > abstract and abstract > concrete contrast (as opposed to concrete > baseline or abstract > baseline contrasts) was chosen in order to avoid a variety of baselines that could range from resting state, fixation cross^51^ to pseudowords^52^ and number or letters^53^ and could affect the interpretation of the results, since subtractions from different baselines create different activation patterns. We acknowledge that this type of contrast does not reveal which brain regions equally support the processing of both concrete and abstract words. The question that this meta-analysis can answer is which are the most replicated data in the literature (in terms of brain activation and words representation) when contrasting abstract and concrete words. Moreover, since concrete and abstract words can dissociate, we aimed at assessing in which anatomical correlates they differ, and not the common ones.

If the same data were reported in different publications, we chose the most recent one and with the highest number of participants^54,55^.

Uncertainties regarding some inclusions were solved by the authors through discussion.

**Figure 1:**
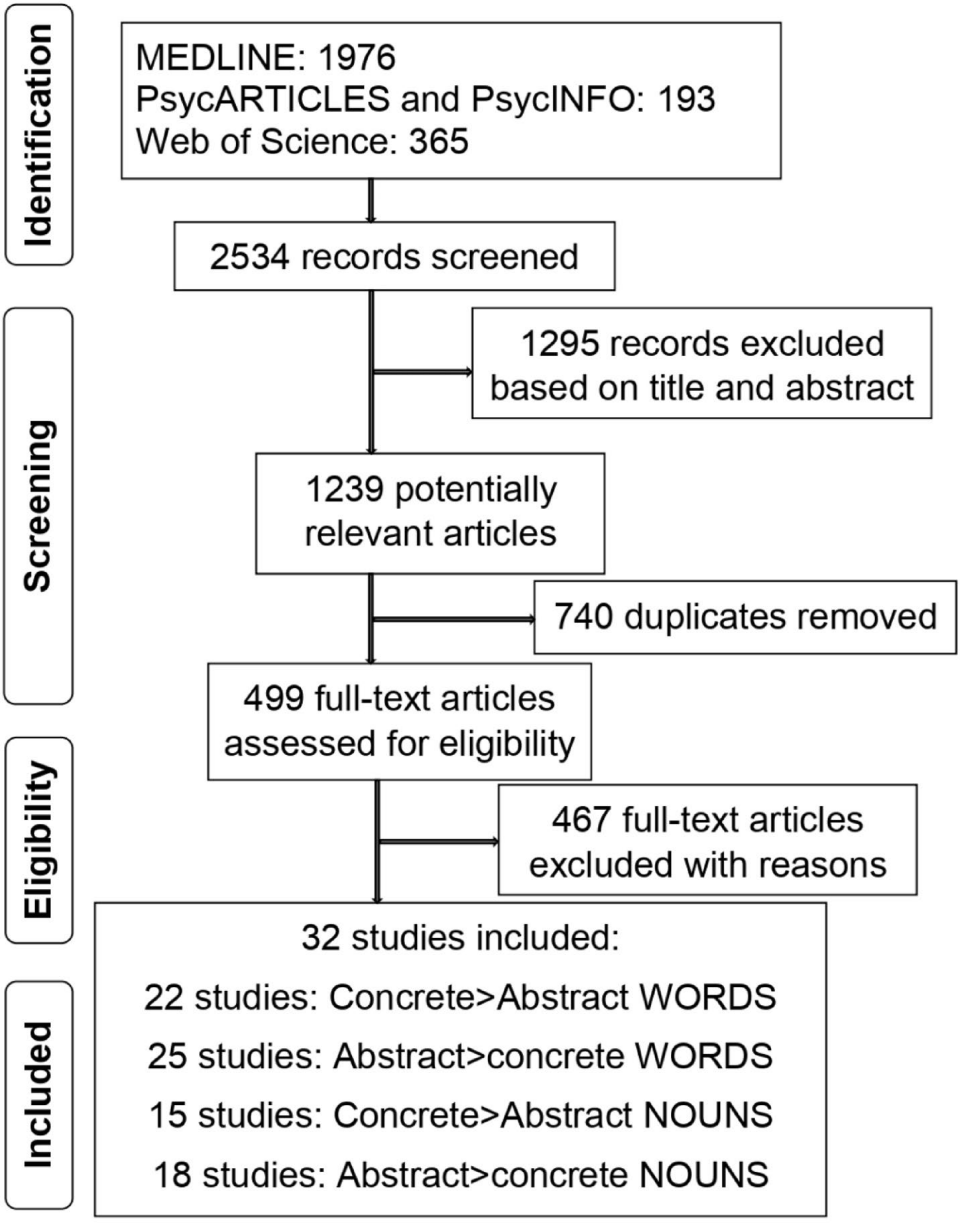
PRISMA flowchart of the selection process for included articles.

The PRISMA flow of information diagram was used to track the search process as presented in Fig. 1 and the main characteristics of the studies included in this meta-analysis are reported in Table 1.

**Table 1:** Descriptive information of the 32 experiments included in the meta-analysis.

	Paper	Technique	Sample size	Age of subjects (years)	Stimuli	Stimuli presentation modality	Experimental task	Design	Random or fixed effect	Contrasts *p* value*
1	D'Esposito et al., 1997	MRI, 1.5 Tesla	7	Range 18–37	English nouns (concrete vs abstract)	Auditory	Mental image generation (concrete) and passive listening (abstract)	blocks	Fixed	*p* < .001 corrected voxel-wise
2	Mellet et al., 1998	PET	8	Range 20–25	French nouns (concrete vs abstract)	Auditory	Mental image generation (concrete) and passive listening (abstract)	blocks	Fixed	*p* = 0.001 uncorrected
3	Perani et al., 1999	PET	14	Range 22–26	Italian words: (1) Concrete verbs, (2) Abstract verbs, (3) Concrete nouns, (4) Abstract nouns	Visual	Lexical decision (classify stimuli as words or nonwords)	Blocks	Fixed	*p* < 0.001 uncorrected
4	Kiehl et al., 1999	MRI, 1.5 Tesla	6	Range 22–26	English words: concrete or abstract	Visual	Lexical decision (classify stimuli as words or nonwords)	Blocks	Fixed	*p* < 0.05 corrected voxel-wise
5	Jessen et al., 2000	MRI, 1.5 Tesla	14	Range 20–44 (31.5 ± 6.3)	German nouns: concrete or abstract	Visual	Memory encoding task	Blocks	Fixed	*p* < 0.001 uncorrected
6	Tyler et al., 2001	PET	9	Range 21–34 26 ± 5	English words: concrete or abstract	Visual	Lexical decision (classify stimuli as words or nonwords)	Block	Fixed-effect	*p* < 0.05 corrected voxel-wise
7	Grossman et al., 2002	MRI, 1.5 Tesla	16	Mean age 23.4	English nouns: animals, implement, abstract	Visual	Semantic judgment (pleasant or not)	Blocks	Fixed	*p* < 0.05 corrected voxel-wise
8	Kounios et al., 2003	MRI, 1.5 Tesla	16	Mean age 73.9	English nouns: animal, implement, and abstract	Visual	Semantic judgement (pleasant or not)	Block	Fixed-effect	*p* < 0.05 corrected
9	Whatmough et al., 2004	PET	15	Range 69–90 74.3 ± 5.6	English nouns: two pairs (concrete or abstract)	Visual	Semantic similarity decision (read aloud if the pairs are similar in meanings)	ns	Ns	*p* < 0.05 corrected voxel-wise
10	Noppeney and Price, 2004	MRI, 2 Tesla	15	Range 21–46 mean age 30	English (1) Abstract concepts, (2) Hand movements, (3) visual attributes (4) Sounds	Visual	Semantic similarity decision	Blocks	Random	*p* < 0.001 uncorrected
11	Fiebach and Friederici, 2004	MRI, 3 Tesla	12	Mean age 25	German nouns: abstract and concrete	Visual	Lexical decision (classify stimuli as words or nonwords)	Event-related	Ns	*p* < 0.05 corrected cluster-wise
12	Giesbrecht et al., 2004	MRI, 1.5 Tesla	10	ns	English words: high imageable and low imageable	Visual	Semantic judgement (words pairs related or unrelated)	Event-related	Random	*p* < 0.005 uncorrected
13	Sabsevitz et al., 2005	MRI, 1.5 Tesla	28	Range 18–33 22.8 ± 3.6	English nouns: concrete and abstract triads	Visual	Semantic similarity decision	Event-related	Random	*p* < .001, uncorrected
14	Binder et al., 2005	MRI, 1.5 Tesla	24	Range 20–50	English nouns: abstract and concrete	Visual	Lexical decision (classify stimuli as words or nonwords)	Event-related	Random	*p* < .005 uncorrected
15	Harris et al., 2006	MRI, 1.5 Tesla	20	Range 19–50 31 ± 9	English nouns: abstract and concrete	Visual	Semantic judgment (positive or negative)	Block	Random	*p* < 0.05 corrected cluster-wise
16	Fliessbach et al., 2006	MRI, 1.5 Tesla	21	Range 19–43 27.4 ± 6.2	German nouns: abstract and concrete	Visual	Recognition task (old/new-decision)	Event-related	Random	*p* < 0.05 corrected cluster-wise
17	Rüschemeyer et al., 2007	MRI, 3 Tesla	20	Range 22–33 27 ± 3	German verbs: simple, complex, motor, abstract	Visual	Lexical decision (classify stimuli as words or nonwords)	Block	Random	*p* < .001, uncorrected
18	Pexman et al., 2007	MRI, 3 Tesla	20	26.5 ± 4.5	English nouns: abstract and concrete	Visual	Semantic categorization (consumable or not)	Event-related	Random	*p* < 0.05 ns
19	Van Dam et al., 2010	MRI, 3 Tesla	16	range 18–38 24 ± 4.63	Dutch verbs denoting (1) Actions that you perform mostly with your arms/hands/ mouth or (2) Abstract events	Visual	Semantic categorization task (go–no go)	Event-related	Random	*p* < 0.05 corrected
20	Zhuang et al., 2011	MRI, 3 Tesla	14	Range 19–33	British English nouns manipulating (cohort competition and imageability)	Auditory	Lexical decision (classify stimuli as words or nonwords)	Event-related	Random	*p* < 0.05 corrected cluster-wise
21	Rodríguez-Ferreiro et al., 2011	MRI, 3 Tesla	14	Range 23–35 mean 29	Spanish verbs: concrete and abstract	Visual	Passive reading	Block	Mixed effects	*p* < .001, uncorrected
22	van Dam et al., 2012	MRI, 3 Tesla	20	Range 18–24 20.5 ± 2.2	Dutch (1) Action color (2) Action nouns (3) Color (4) Abstract nouns	Auditory	Semantic categorization (action or color characteristics)	Block	Random	*p* < 0.005 ns
23	Wilson-Mendenhall et al., 2013	MRI, 3 Tesla	13	Range 18–24	English words: two abstract (convince, arithmetic) two concrete (rolling, red)	Visual	Semantic task (concept–scene match)	Block	Random	*p* < 0.05 corrected voxel-wise
24	Vigliocco et al., 2013	MRI, 3 Tesla	20	Range 18–33 21.9 ± 4.4	English nouns: abstract and concrete	Visual	Lexical decision (classify stimuli as words or nonwords)	Block	Random	*p* < 0.05 FWE-cluster-wise
25	Hayashi et al., 2014	MRI, 1.5 Tesla	16	Range 20–36 26.1 ± 5.9	Japanese kanji nouns: concrete and abstract	Visual	Generate visual imagery	Block	Random	*p* < .001, uncorrected
26	Roxbury et al., 2014	MRI, 3 Tesla	17	27 ± 5.1	English nouns: concrete, abstract and pseudowords	Auditory	Lexical decision (classify stimuli as words or nonwords)	Event-related	Random	*p* < .001, uncorrected
27	Skipper and Olson, 2014	MRI, 3 Tesla	19	Mean age 23	English nouns: concrete and abstract	Visual	Semantic task (answer to question in reference to the 3 words in the block)	Block	Ns	*p* < 0.001 FDR corrected cluster-wise
28	Hoffman et al., 2015	MRI, 3 Tesla	20	Range 20–39 mean: 25	English words: concrete and abstract	Visual	Semantic task (synonym judgement)	Block	Random	*p* < 0.05 corrected cluster-wise
29	Kumar, 2016	MRI, 3 Tesla	20	28.3 ± 3	Hindi nouns: abstract, concrete and non-words	Visual	Perceptual task (orthography judgment)	Block	Fixed	*p* < 0.05 corrected
30	Wang et al., 2019	MRI, 3 Tesla	23	Range 19–29 mean 22.17	Chinese nouns: abstract, concrete	Visual	Semantic task (which of the choices was more semantically related to the probe)	Block	Ns	*p* < 0.05 FWE corrected cluster-level
31	Pauligk et al., 2019	MRI, 3 Tesla	21	23.3 ± 1.9	German nouns: abstract and concrete	Visual	Delayed lexical decision task (classify stimuli as words or nonwords)	Block	Ns	*p* = 0.001 corrected voxel-wise
32	Meersmans et al., 2020	MRI, 3 Tesla	26	Range 18–34 22.9 ± 3.7	Dutch nouns: abstract and concrete	Visual and auditory	Overt repetition task	Event-related	Random	*p* < 0.001 uncorrected *p* < 0.05 FWE-corrected

Age is reported in years and when it was specified means and standard deviations are presented.

p values (the statistical threshold for the neuroimaging univariate analysis conducted in the included papers) are reported as they were presented in the original articles; the exact value and the correction procedure was not always specified.

ns, not specified.

### Classification of the raw data before clustering analyses

From the selected papers, only the stereotactic coordinates representing the concrete > abstract or abstract > concrete contrasts were extracted. Following this procedure, we obtained 295 foci from a total sample of 535 participants. The stereotaxic coordinates reported in terms of the Talairach and Tournoux atlas^56^ were transformed into the MNI (Montreal Neurological Institute) stereotaxic space^57^ using the tal2icbm transforms implemented in the GingerALE software^41,43,58^.

For all the stereotaxic coordinates we extracted the relevant information about the statistical comparisons that generated them. More explicitly, we reported the MNI coordinates (MNI x,y,z), the name of the first author, the journal and the year of publication of the paper, the technique (PET or fMRI) and the stereotactic space used, the age of participants, the type of task, the nature of the contrast from which the peak was extracted, the statistical thresholds, the stimulus type (nouns or verbs) and the presentation modality (auditory or visual).

### Clustering procedure

Once obtained the set of MNI coordinates, the meta-analyses were carried out using the revised ALE algorithm^41,43^ implemented into GingerALE software Version 3.0.2^58^ (http://brainmap.org/ale). The ALE algorithm aims to identify areas with a convergence of reported coordinates across experiments that are higher than expected from a random spatial association. The logic behind this approach implies a spatial probability distribution modeled for each activation peak included in the dataset of interest. Reported foci are treated as centers of 3D Gaussian probability distributions capturing the spatial uncertainty associated with each focus^58^. The between-subject variance is weighted by the number of participants per study, since larger sample sizes should provide more reliable approximations of the “true” activation effect. The voxel-by-voxel union of these distributions is used as an activation likelihood map, subsequently tested for statistical significance against randomly generated sets of foci. ALE was proven to be a reliable way of blending evidence from multiple studies^43^ and was used successfully in different fields e.g.,^59^.

More specifically we used the following procedure:

- Anatomical filtering—we applied a first filtering of the coordinates using the most conservative (smallest) mask available in the GingerALE software and 17 foci from the total of 295 fell out of the mask.
- ALE maps (quantify the degree of overlap in peak activation across experiments) were calculated using the modified ALE algorithm and the random-effects model^41,43^;
- Thresholding procedure—for each ALE calculation described below significance was tested using 1000 permutations with a cluster forming threshold of *p* < 0.001 (uncorrected). In order to increase test sensitivity to false positives significance was corrected with a cluster-level family-wise error threshold of *p* < 0.05^46^ as used by other meta-analytic studies^60^.

Unfortunately, ALE cannot deal with multiple independent variables designs, and in this paper we intended to consider the role of different variables like (1) stimulus type (nouns only, verbs only or all word stimuli), (2) modality of presentation (visual only, auditory only or both visual and auditory), and (3) task specificity (e.g., lexical, semantic tasks or all tasks). The ALE strategy we choose in this case was to consider separate sets of foci for each variable and run one meta-analysis for each of these sets when the number of papers was large enough. To this purpose, the overall dataset was divided a-posteriori into several subsets, which automatically implied running meta-analyses on a low number of foci (lowering the power). An important limitation of this approach is that we are not able to statistically assess the interaction between variables like stimuli type and task.

The analyses were based on the following contrasts:

1. An analysis included the activation peaks associated with **word processing** independently of the stimulus type and task
  *concrete words* > *abstract words* included 149 stereotactic activation loci from 22 studies, 353 participants (8 foci out of mask)^51,53,54,61–79^;
  *abstract words* > *concrete words* included 146 stereotactic activation loci from 25 studies, 415 participants (9 foci out of mask)^51–53,61–64,67–70,73–75,77,78,80–88^;
2. An analysis with peaks associated with **noun processing** only (because the number of studies including verbs only was too small (4 studies) for a specific analysis on this type of stimuli^70,76,82,83^)
  *concrete nouns* > *abstract nouns* included 107 stereotactic activation loci from 15 studies (5 foci out of mask), 251 participants;
  *abstract nouns* > *concrete nouns* included 99 stereotactic activation loci from 18 studies (8 foci out of mask), 324 participants;
3. An analysis included the activation peaks associated with word processing independently of the stimulus type (verbs, names or adjectives), but taking into consideration only **visually presented stimuli**
  *concrete words* > *abstract words visual stimuli only* included 121 stereotactic activation loci from 18 studies, 301 participants
  *abstract words* > *concrete words visual stimuli only* included 135 stereotactic activation loci from 22 studies, 374 participants

Since only 5 studies included auditory stimuli we could not perform a specific analysis for this category^51,54,62,79,89^.

(4) An analysis on peaks associated with **lexical** (words or non-words classification task), or **semantic decision tasks** (e.g., pleasantness decision task, answering a question about the stimuli), excluding all the studies based on: memory tasks (2 studies), perceptual decision task (1 study), mental image generation (3 studies), passive reading (2 studies).
  *concrete* > *abstract word (only lexical and semantic tasks)* included 114 stereotactic activation loci from 16 studies, 273 participants
  *abstract* > *concrete word (only lexical and semantic tasks)* included 116 stereotactic activation loci from 17 studies, 289 participants

We explored a-posteriori the role of the (1) stimulus type (nouns), (2) modality of presentation (visual stimuli), and (3) task specificity (lexical and semantic tasks) in order to control as much as possible for each of these variables, i.e., to increase the results accuracy.

It might be argued against the inclusion of PET and fMRI studies in the same meta-analysis due to the substantial methodological differences between the two techniques in terms of experimental design, processing, spatial localization and cluster accuracy. Because the number of studies investigating concrete vs. abstract words is small, we decided to include data from both techniques in order to increase power in the analyses. Nevertheless, in Appendix A (supplementary materials), we present the data analysis after excluding all PET studies (figures and tables are numbered as e.g., Fig. 1A and Table 1A).

For anatomical labeling and figures, we capitalized on the Automatic Anatomical Labeling (AAL) template available in the MRIcron visualization Software (https://www.nitrc.org/projects/mricron).

## Results

Once the appropriate studies were collected, we used activation likelihood estimation (ALE) to meta-analytically remodel available neuroimaging data.

### CONCRETE > ABSTRACT meta-analysis

**Figure 2:**
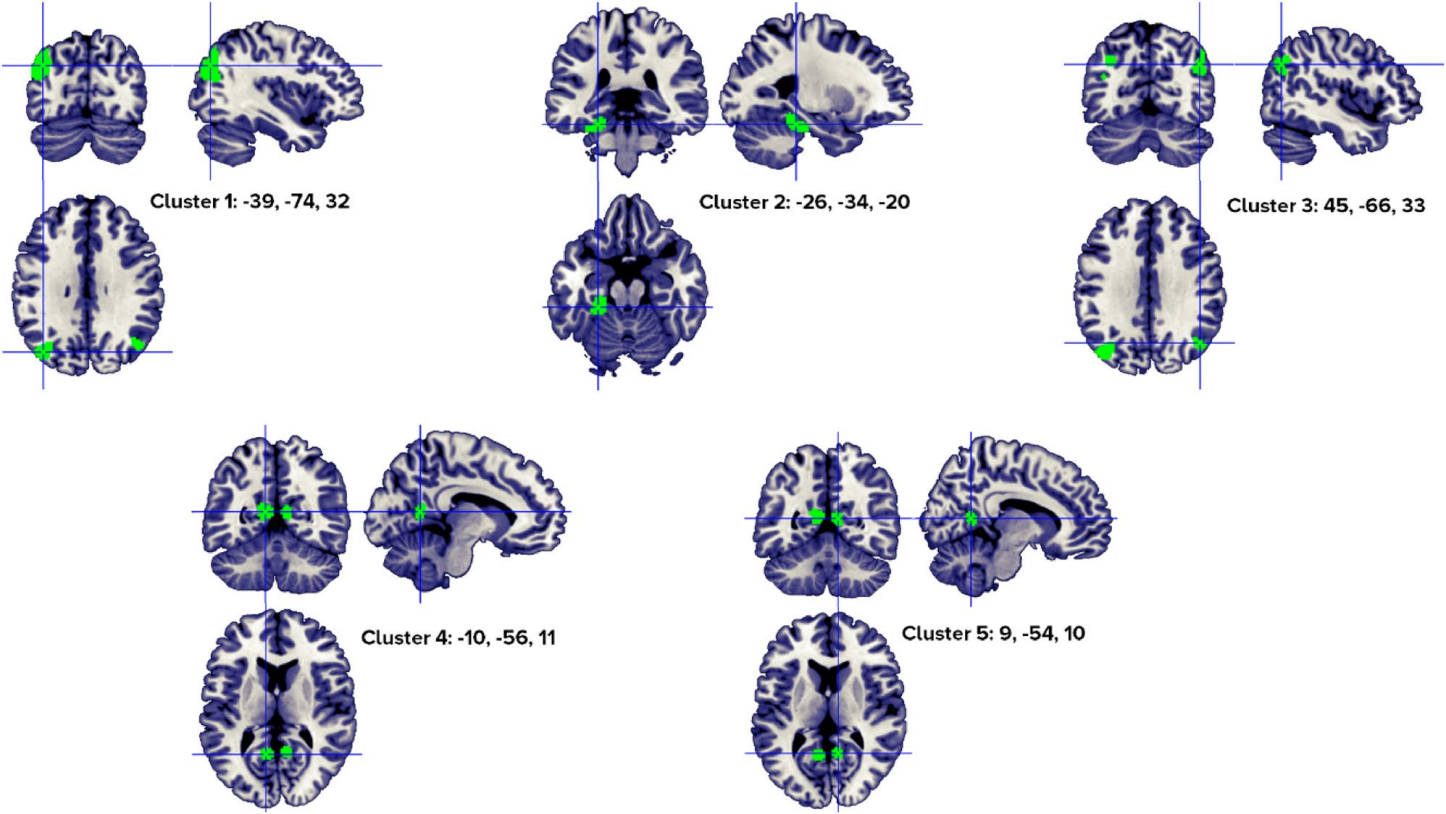
Clusters activated by the concrete > abstract words contrast. The crosses are centered in the areas correspond to stereotactic coordinates reported in Table 2. The images are presented in neurological convention.

The GingerALE procedure run over the concrete words > abstract words set of coordinates identified a total of 5 clusters, with 1–4 individual peaks each, from 4 to 11 different studies (Fig. 2). Regions that were consistently activated across experiments were localized in the bilateral middle temporal gyrus and posterior cingulate, the left parahippocampal gyrus, left fusiform gyrus, bilateral precuneus and angular gyri, left superior occipital gyrus and left cerebellum culmen. The peaks distribution for each significant cluster is reported in Table 2.

**Table 2:** Concrete > abstract word clusters.

H	Cluster	Macroanatomical location		Cytoarchitectonic Label	Weighted center (MNI; mm)			Vol. (mm^3^)	Peaks: MNI coordinates (mm)			ALE score	Z Score	Nr	Contributors to cluster
		Lobe	Gyrus		x	y	Z		x	y	z				Studies
L	1	Temporal Occipital Parietal	Superior Occipital Middle Temporal Precuneus, Angular Cuneus	BA 39, BA 19	− 38.6	− 74.2	31.8	4680	− 40	− 74	34	0.025	5.859	11	Jessen, 2000 (1); Sabsevitz, 2005 (3); Binder, 2005 (1); Harris, 2006 (1); van Dam, 2010 (1); Zhuang, 2011 (1); Rodríguez-Ferreiro, 2011 (2); van Dam, 2012 (1); Roxbury, 2014 (1); Skipper, 2014 (5); Hoffman, 2015 (2)**
									− 44	− 78	24	0.020	4.978		
									− 40	− 70	22	0.015	4.117		
									− 38	− 72	46	0.014	4.048		
L	2	Cerebellum Anterior Lobe, Limbic Lobe Temporal	Culmen (cerebellum), Parahippocampal Fusiform	BA 35, BA 36	− 25.9	− 34.3	− 19.8	2584	− 24	− 36	− 18	0.021	5.262	7	Sabsevitz, 2005 (2); Harris, 2006 (1); Rodríguez-Ferreiro, 2011 (2); van Dam, 2012 (1); Hayashi, 2014 (1); Roxbury, 2014 (1); Hoffman, 2015 (2)**
									− 24	− 30	− 22	0.019	4.928		
									− 34	− 36	− 24	0.013	3.764		
R	3	Parietal Temporal	Inferior Parietal Angular Precuneus, Middle Temporal	BA 39	44.8	− 65.6	33.4	1648	44	− 68	32	0.017	4.496	4	Sabsevitz, 2005 (2); van Dam, 2010 (1); Roxbury, 2014 (1); Hoffman, 2015 (2)**
									42	− 60	36	0.012	3.640		
									48	− 66	44	0.012	3.547		
L	4	Limbic Occipital	Posterior Cingulate Lingual Gyrus Cuneus	BA 30, BA 18	− 10.0	− 56.1	11.3	1184	− 10	− 56	12	0.018	4.756	5	Sabsevitz, 2005 (1); Binder, 2005(1); Harris, 2006(1); Rüschemeyer, 2007(1); Roxbury, 2014(1)**
R	5	Limbic Lobe	Posterior Cingulate	BA 30	8.5	− 54.0	10.0	840	8	− 54	10	0.019	4.891	4	Sabsevitz, 2005 (1); Harris, 2006 (1); Rodríguez-Ferreiro, 2011 (1);Hoffman, 2015 (1)**

Included 149 stereotactic activation loci from 22 studies, 353 participants, Chosen min. cluster size 736 mm^3^.

All the values and labels were extracted from the GingerALE output files. Clusters are ordered for decreasing volume size. Coordinates (x, y, z) are in the MNI space.

H = Hemisphere; ALE = activation likelihood estimation; Nr. = number of studies that contributed to each cluster; L = left; BA = Brodmann area; ** = between brackets are the number of foci from each study that contributed to that specific cluster; R = right.

**Figure 3:**
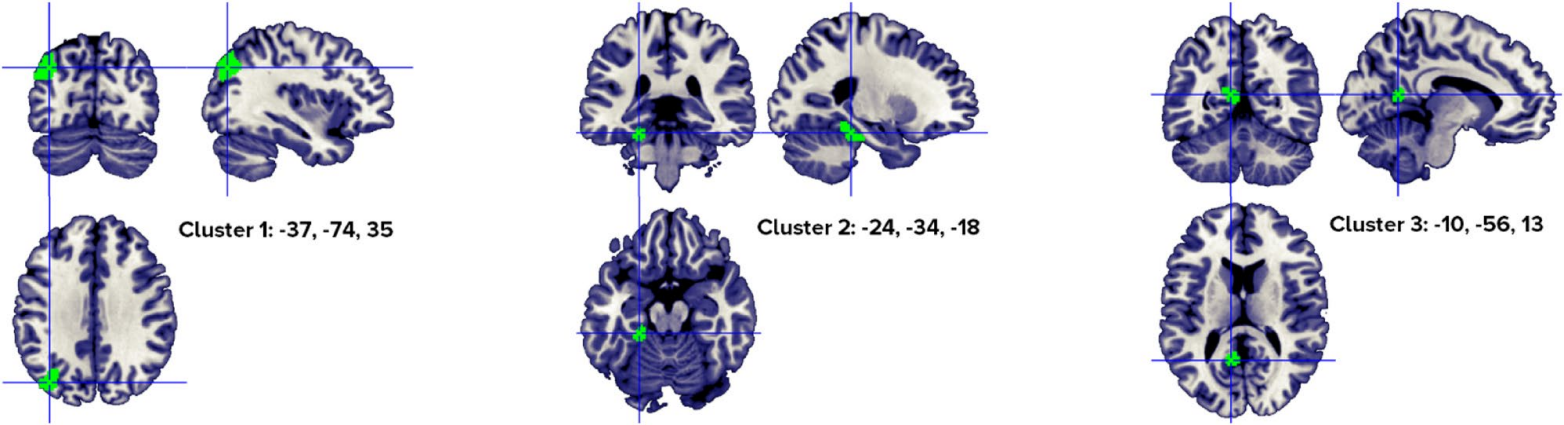
Clusters activated by the concrete > abstract nouns contrast. The crosses are centered in the areas correspond to stereotactic coordinates reported in Table 3. The images are presented in neurological convention.

A similar activation pattern, except for the right hemisphere involvement, was observed when only studies reporting exclusively noun stimuli were taken into consideration (concrete nouns > abstract nouns). We observed three left activation clusters (Fig. 3, Table 3) situated in the middle temporal gyrus, parahippocampal gyrus, posterior cingulate, precuneus, superior occipital gyrus, and culmen (left cerebellum anterior lobe).

**Table 3:** Concrete > abstract nouns clusters.

H	Cluster	Macroanatomical location		Cytoarchitectonic Label	Weighted center (MNI; mm)			Vol. (mm^3^)	Peaks: MNI coordinates (mm)			ALE score	Z Score	No	Contributors to cluster
		Lobe	Gyrus		x	Y	z		x	y	z				Studies
L	1	Occipital, Parietal, Temporal	Superior Occipital, Precuneus, Middle Temporal, Angular	BA 39, BA 19	− 37.1	− 73.8	35.2	3376	− 34	− 68	36	0.019	5.112	8	Jessen, 2000 (1); Sabsevitz, 2005 (3); Binder, 2005 (1); Harris, 2006 (1); Zhuang, 2011 (1); van Dam, 2012 (1); Roxbury, 2014 (1); Skipper, 2014 (4)**
									− 38	− 74	32	0.019	5.051		
									− 34	− 78	38	0.017	4.784		
									− 46	− 76	28	0.015	4.320		
									− 38	− 72	46	0.014	4.240		
L	2	Anterior, Limbic	Culmen, Parahippocampal	BA 36, BA 35	− 23.7	− 34.2	− 18.3	1432	− 24	− 36	− 18	0.015	4.392	4	Hayashi, 2004 (1); Sabsevitz, 2005 (2); Harris, 2006 (2); Roxbury, 2014 (1)**
L	3	Limbic, Occipital	Posterior Cingulate, Cuneus	BA 30, BA 29	− 9.5	− 55.7	12.5	1040	− 10	− 56	12	0.017	4.745	4	Sabsevitz, 2005 (1); Binder, 2005 (2); Harris, 2006 (1); Roxbury, 2014 (1)**
									− 8	− 46	14	0.009	3.338		

Included 107 stereotactic activation loci from 15 studies, 251 participants, Chosen min. cluster size 720 mm^3^.

All the values and labels were extracted from the GingerALE output files. Clusters are ordered for decreasing volume size. Coordinates (x, y, z) are in the MNI space.

H = Hemisphere; ALE = activation likelihood estimation; Nr. = number of studies that contributed to each cluster; L = left; BA = Brodmann area; ** = between brackets are the number of foci from each study that contributed to that specific cluster.

**Figure 4:**
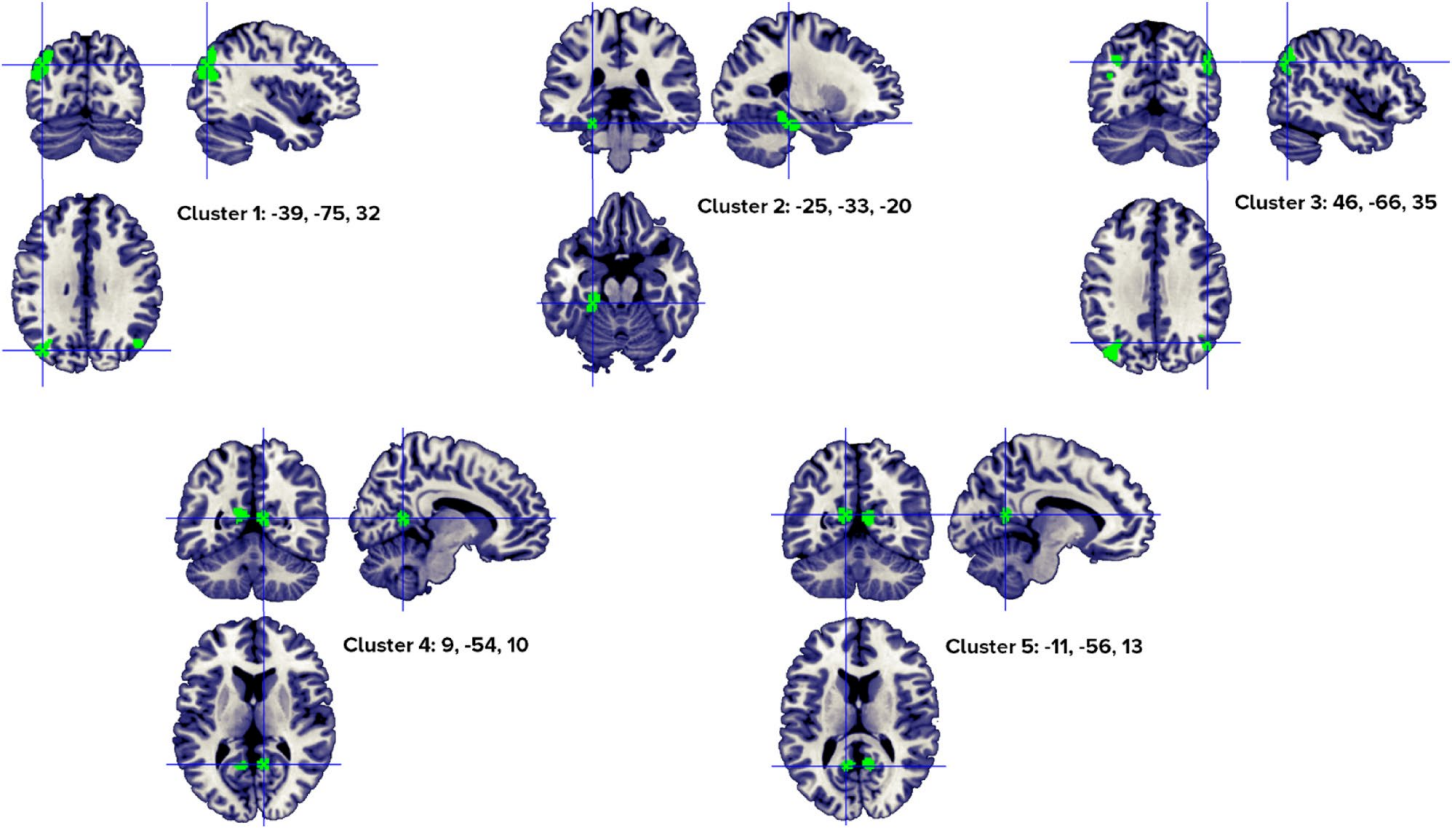
Clusters activated by the concrete > abstract words—visual stimuli—contrast. The crosses are centered in the areas correspond to stereotactic coordinates reported in Table 4. The images are presented in neurological convention.

The ALE procedure run over the concrete words > abstract words, visual stimuli only set of coordinates, identified a total of 5 clusters, with 1–6 individual peaks each, from 4 to 8 different studies (Fig. 4). Regions that were consistently activated across experiments were localized in the left middle temporal gyrus, bilateral posterior cingulate, and parahippocampal gyrus, left fusiform gyrus, bilateral precuneus and angular gyri, left superior occipital gyrus and left cerebellum culmen. The peaks distribution for each significant cluster is reported in Table 4.

**Table 4:** Concrete > abstract words—visual stimuli- clusters.

H	Cluster	Macroanatomical location		Cytoarchitectonic Label	Weighted center (MNI; mm)			Vol. (mm^3^)	Peaks: MNI coordinates (mm)			ALE score	Z Score	No	Contributors to cluster
		Lobe	Gyrus		x	Y	z		x	y	z				Studies
L	1	Temporal, Occipital, Parietal	Superior Occipital, Middle Temporal, Precuneus, Angular	BA 19, BA 39	− 38.5	− 74.6	31.9	4360	− 40	− 76	34	0.0212	5.3445	8	Sabsevitz, 2005 (1); Binder, 2005 (3); Harris, 2006 (1); van Dam, 2010 (1); Zhuang, 2011 (1); Rodríguez-Ferreiro, 2011 (2); Skipper, 2014 (5); Hoffman, 2015 (2)**
									− 44	− 78	24	0.0174	4.7222		
									− 36	− 78	38	0.0172	4.6952		
									− 34	− 68	36	0.0169	4.6447		
									− 40	− 70	22	0.0145	4.1705		
									− 38	− 72	46	0.0143	4.1460		
L	2	Limbic Lobe, Anterior, Temporal	Parahippocampal, Culmen, Fusiform	BA 35, BA 36	− 25	− 33.2	− 19.5	1752	− 24	− 30	− 22	0.0180	4.8145	4	Sabsevitz, 2005 (2); Harris, 2006 (1); Hayashi, 2014 (1); Hoffman, 2015 (2)**
									− 26	− 38	− 16	0.0169	4.6525		
R	3	Parietal	Inferior Parietal, Angular, Precuneus	BA 39	45.9	− 65.9	35	1336	46	− 68	34	0.0151	4.2998	4	Jessen, 2000 (1); Sabsevitz, 2005 (2); van Dam, 2010 (1); Hoffman, 2015 (2)**
									48	− 66	44	0.0118	3.6536		
									42	− 60	36	0.0116	3.6238		
R	4	Limbic	Posterior Cingulate, Parahippocampal	BA 30, BA 29	8.7	− 54	10	992	8	− 54	10	0.0192	5.0153	4	Sabsevitz, 2005 (1); Harris, 2006 (1); Rodríguez-Ferreiro, 2011 (1); Hoffman, 2015 (1)**
L	5	Limbic, Occipital	Posterior Cingulate, Lingual	BA 30, BA 18	− 11	− 55.6	12.8	888	− 12	− 56	14	0.0164	4.5739	4	Sabsevitz, 2005 (1); Binder, 2005 (1); Harris, 2006 (1); Rüschemeyer, 2007 (1)**

Included 121 stereotactic activation loci from 18 studies, 301 participants, chosen min. cluster size 616 mm^3^.

All the values and labels were extracted from the GingerALE output files. Clusters are ordered for decreasing volume size. Coordinates (x, y, z) are in the MNI space.

H = Hemisphere; ALE = activation likelihood estimation; Nr. = number of studies that contributed to each cluster; L = left; BA = Brodmann area; ** = between brackets are the number of foci from each study that contributed to that specific cluster; R = right.

**Figure 5:**
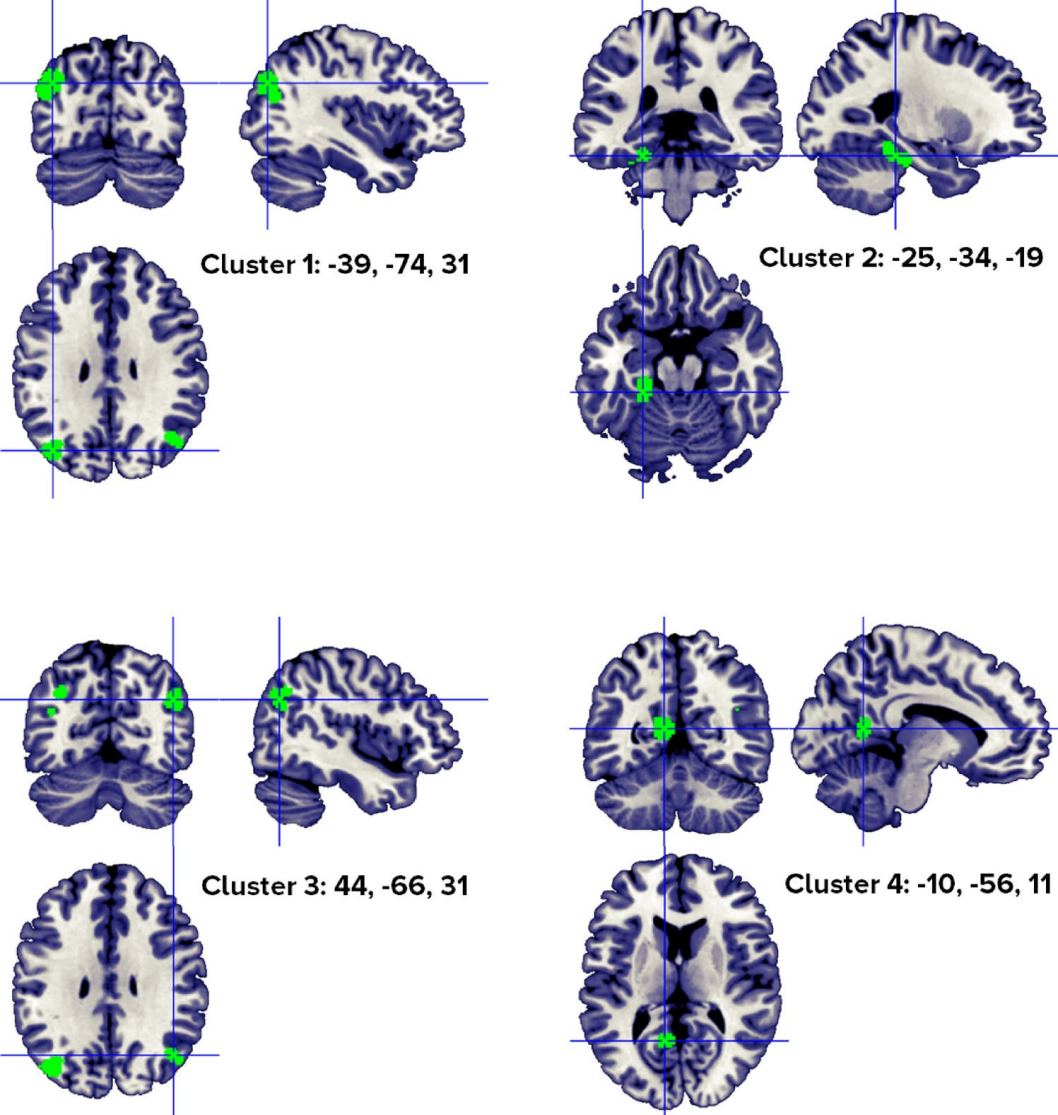
Clusters activated by the concrete > abstract words -semantic and lexical tasks—contrast. The crosses are centered in the areas correspond to stereotactic coordinates reported in Table 5. The images are presented in neurological convention.

A comparable activation pattern was observed when only studies based on lexical and semantic tasks were taken into consideration. The analysis indicated 4 activation clusters correlated with concrete words > abstract words—lexical and semantic tasks: bilateral middle temporal gyrus, left posterior cingulate and the left parahippocampal gyri, bilateral precuneus, left angular, left superior occipital gyrus and left cerebellum culmen (Fig. 5, Table 5).

**Table 5:** Concrete > abstract words—semantic and lexical tasks only- clusters.

H	Cluster	Macroanatomical location		Cytoarchitectonic label	Weighted center (MNI; mm)			Vol. (mm^3^)	Peaks: MNI coordinates (mm)			ALE score	Z Score	Nr	Contributors to cluster
		Lobe	Gyrus		X	y	z		x	y	z				Studies
L	1	Occipital, temporal, parietal	Superior occipital, middle temporal, precuneus, angular	BA 19, BA 39	− 38.6	− 73.9	31.3	3856	− 40	− 74	34	0.0247	5.9191	9	Sabsevitz, 2005 (2); Binder, 2005 (1); Harris, 2006 (1); van Dam, 2010 (1); Zhuang, 2011 (1); van Dam, 2012 (1); Roxbury, 2014 (1), Skipper, 2014 (5); Hoffman, 2015 (2)**
									− 46	− 78	26	0.0183	4.8754		
									− 40	− 70	22	0.0144	4.1581		
L	2	Limbic Lobe, Anterior lobe, Temporal	Parahippocampal, Culmen	BA 35, BA 36	− 24.9	− 33.8	− 18.6	1792	− 24	− 38	− 16	0.0193	5.0320	5	Sabsevitz, 2005 (2); Harris, 2006 (1); van Dam, 2012 (1); Roxbury, 2014 (1); Hoffman, 2015 (2)**
									− 24	− 28	− 22	0.0156	4.4119		
R	3	Temporal, Parietal	Middle Temporal, Precuneus	BA 39	44.3	− 65.6	31.4	1696	44	− 68	32	0.0167	4.6298	4	Sabsevitz, 2005 (2); van Dam, 2010 (1); Roxbury, 2014 (1); Hoffman, 2015 (3)**
									42	− 60	36	0.0122	3.7327		
									40	− 56	24	0.0091	3.1937		
L	4	Limbic Lobe, Occipital	Posterior Cingulate, Lingual	BA 30, BA 18	− 10.1	− 55.9	11.4	1392	− 10	− 56	12	0.0184	4.8883	5	Sabsevitz, 2005 (1); Binder, 2005 (2); Harris, 2006 (1); Rüschemeyer, 2007 (1); Roxbury, 2014 (1)**
									− 8	− 46	14	0.0095	3.2622		

Included 114 stereotactic activation loci from 16 studies, 273 participants, chosen min. cluster size 656 mm^3^.

All the values and labels were extracted from the GingerALE output files. Clusters are ordered for decreasing volume size. Coordinates (x, y, z) are in the MNI space.

H = Hemisphere; ALE = activation likelihood estimation; Nr. = number of studies that contributed to each cluster; L = left; BA = Brodmann area; ** = between brackets are the number of foci from each study that contributed to that specific cluster; R = right.

### Abstract > concrete meta-analysis

**Figure 6:**
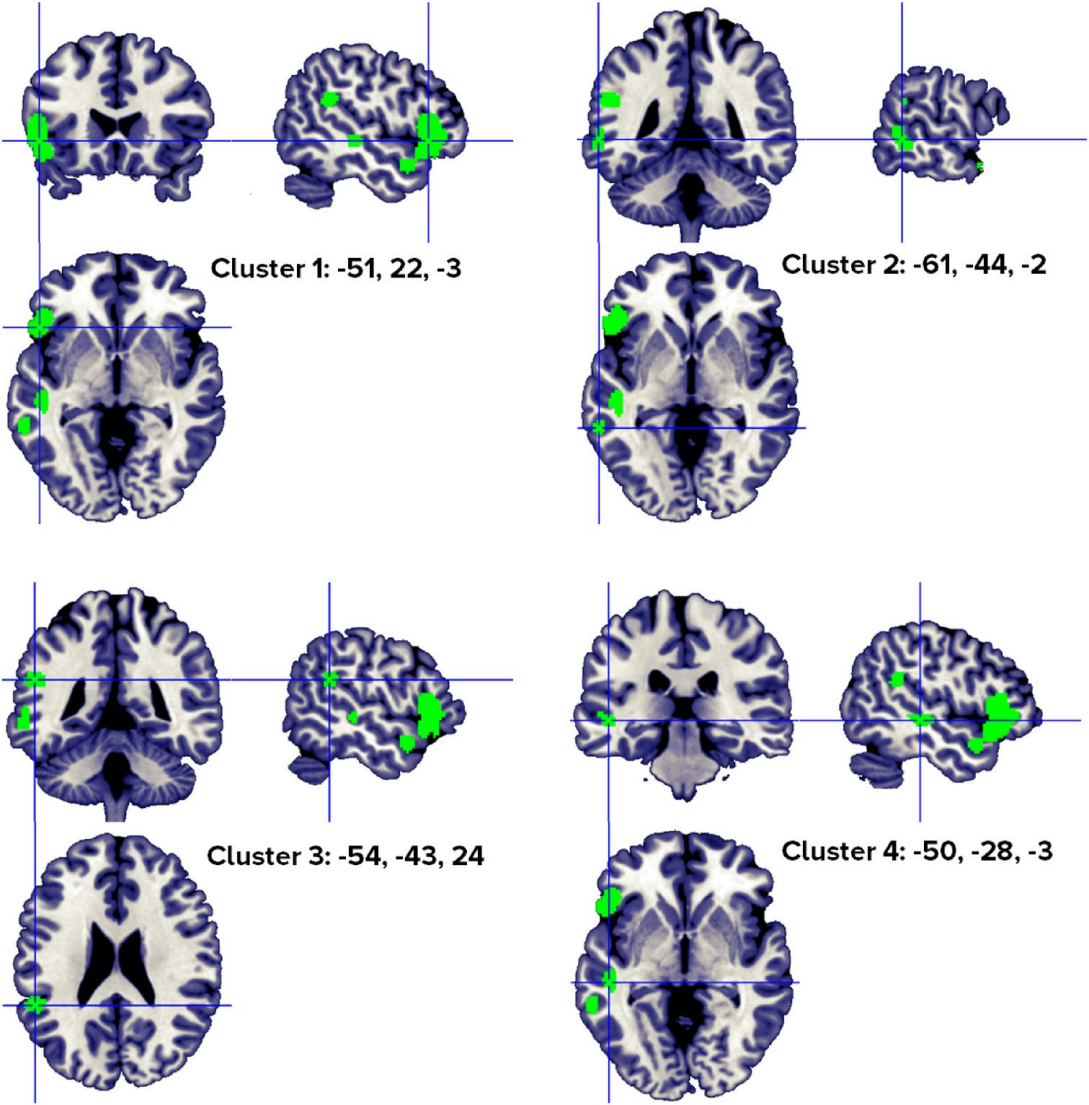
Clusters activated by the abstract > concrete words contrast. The crosses are centered in the areas correspond to stereotactic coordinates reported in Table 6. The images are presented in neurological convention.

The revised ALE algorithm discriminated four clusters that correlated with abstract word processing in a healthy population (Fig. 6), from four to 12 different papers (Table 6). Our analyses identified a robust neural pattern of activity in the left frontal and temporal lobes, specifically, the inferior frontal gyrus, the superior and middle temporal gyri and left inferior parietal.

**Table 6:** Abstract > concrete word clusters.

H	Cluster	Macroanatomical location		Cytoarchitectonic Label	Weighted center (MNI; mm)			Vol. (mm^3^)	Peaks: MNI coordinates (mm)			ALE score	Z Score	Nr	Contributors to cluster
		Lobe	Gyrus		x	y	Z		x	y	z				Studies
L	1	Frontal, Temporal	Inferior Frontal, Superior Temporal	BA 45, BA 47, BA 44	− 50.8	21.7	− 3.4	6680	− 52	24	4	0.029	6.369	12	Perani, 1999 (2); Fiebach, 2004 (1); Sabsevitz, 2005 (3); Binder, 2005 (5); Fliessbach, 2006 (2); Pexman, 2007 (1); Rodríguez-Ferreiro, 2011 (2); Hayashi, 2014 (1); Hoffman, 2015 (3); Skipper, 2014 (2); Wang, 2019 (1); Pauligk, 2019 (2)**
									− 48	20	− 10	0.025	5.835		
									− 54	8	− 18	0.016	4.346		
L	2	Temporal	Middle Temporal	BA 22, BA 21	− 60.5	− 44.4	− 1.6	1048	− 60	− 42	− 6	0.016	4.369	5	Noppeney, 2004 (1); Sabsevitz, 2005 (1); Pexman, 2007 (2); Rodríguez-Ferreiro, 2011 (2); Wang, 2019 (1)**
									− 60	− 48	4	0.015	4.219		
L	3	Temporal Parietal	Superior Temporal Inferior Parietal,	BA 13, BA 40	− 53.5	− 42.9	24.1	960	− 54	− 42	24	0.021	5.112	4	Hayashi, 2014 (1); Hoffman, 2015 (2); Wang, 2019 (1); Meersmans, 2020 (1)**
L	4	Temporal	Superior and Middle Temporal	BA 22, BA 21	− 49.5	− 27.5	− 2.6	840	− 50	− 28	− 4	0.016	4.350	4	Sabsevitz, 2005 (1); Hoffman, 2015 (2); Kumar, 2016 (1); Wang, 2019 (1)**

Included 146 stereotactic activation loci from 25 studies, 415 participants, chosen min. cluster size 704 mm^3^.

All the values and labels were extracted from the GingerALE output files. Clusters are ordered for decreasing volume size. Coordinates (x, y, z) are in the MNI space.

H = Hemisphere; ALE = activation likelihood estimation; Nr. = number of studies that contributed to each cluster; L = left; BA = Brodmann area; ** = between brackets are the number of foci from each study that contributed to that specific cluster.

**Figure 7:**
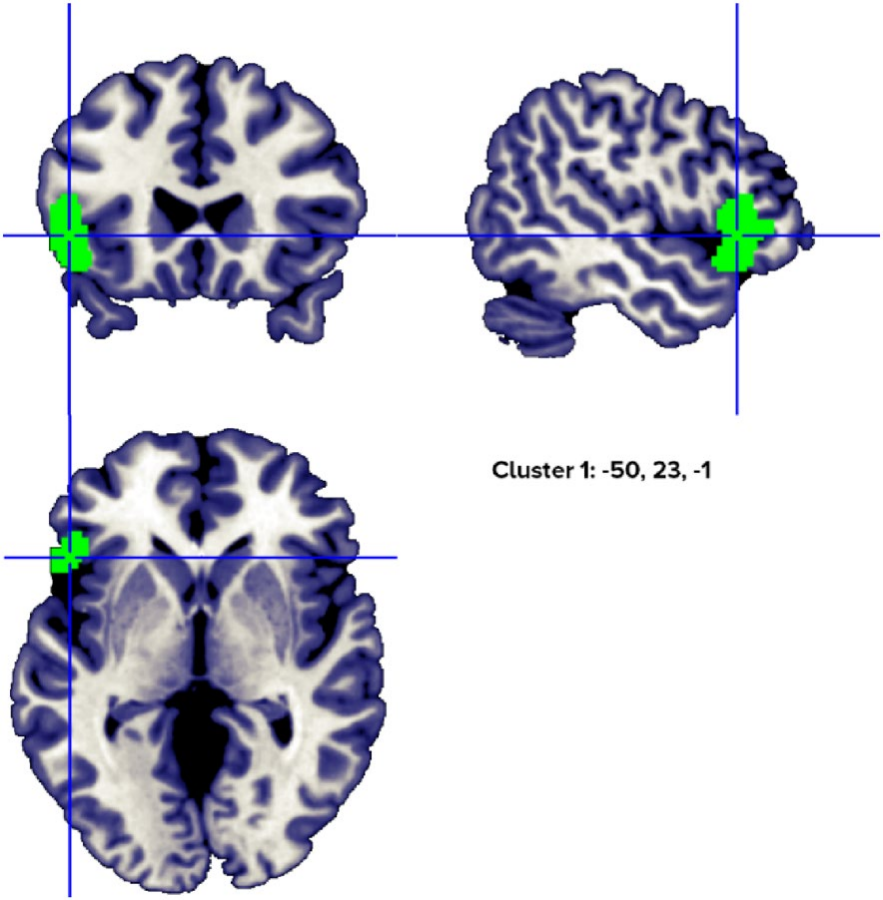
Clusters activated by the abstract > concrete nouns contrast. The crosses are centered in the areas correspond to stereotactic coordinates reported in Table 7. The images are presented in neurological convention.

When only abstract nouns (abstract nouns > concrete nouns) were analyzed, the results indicated a single cluster with two peaks, from 9 studies, in the left inferior frontal gyrus (Fig. 7, Table 7).

**Table 7:** Abstract > concrete nouns clusters.

H	Cluster	Macroanatomical location		Cytoarchitectonic label	Weighted Center (MNI; mm)			Vol. (mm^3^)	Peaks: MNI Coordinates (mm)			ALE score	Z score	Nr	Contributors to cluster
		Lobe	Gyrus		X	y	z		x	y	z				Studies
L	1	Frontal	Inferior Frontal, Precentral	BA 47, BA 45, BA 44	− 50.1	23.4	− 1.3	4520	− 52	22	6	0.0253	6.118	9	Fiebach, 2004 (1); Sabsevitz, 2005 (2); Binder, 2005 (3); Fliessbach, 2006 (2); Pexman, 2007 (1); Hayashi, 2004 (1); Skipper, 2014 (2); Wang, 2019 (1); Pauligk, 2019 (2)**
									− 48	22	− 10	0.0227	5.712		

Included 99 stereotactic activation loci from 18 studies, 324 participants, chosen min. cluster size 728 mm^3^.

All the values and labels were extracted from the GingerALE output files. Clusters are ordered for decreasing volume size. Coordinates (x, y, z) are in the MNI space.

H = Hemisphere; ALE = activation likelihood estimation; Nr. = number of studies that contributed to each cluster; L = left; BA = Brodmann area; ** = between brackets are the number of foci from each study that contributed to that specific cluster.

**Figure 8:**
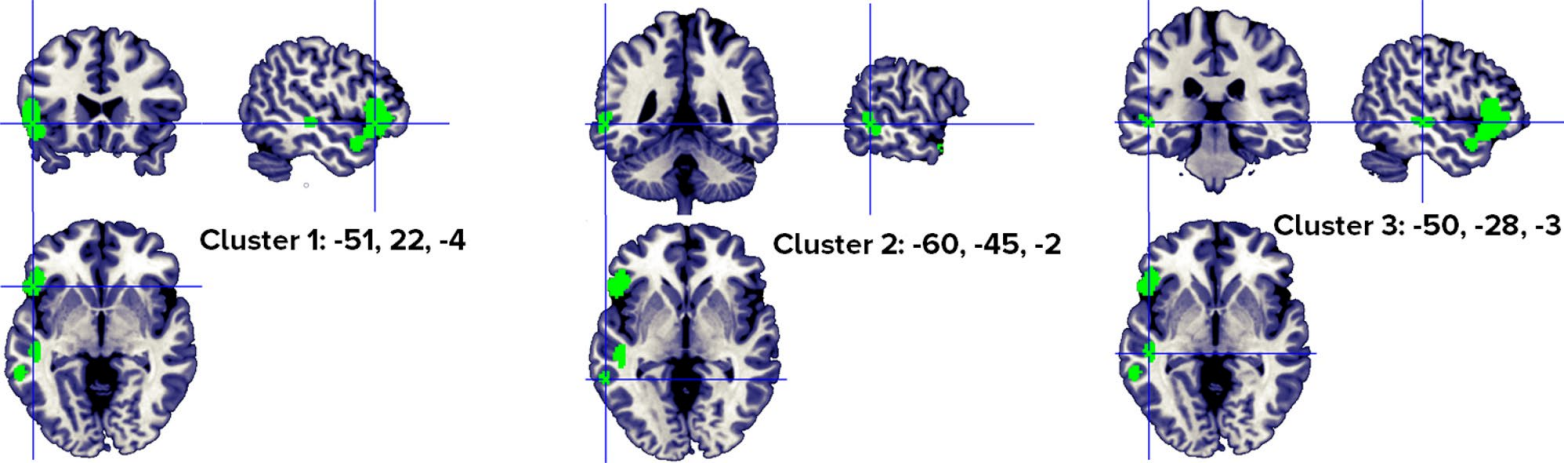
Clusters activated by the abstract > concrete words—visual stimuli—contrast. The crosses are centered in the areas correspond to stereotactic coordinates reported in Table 8. The images are presented in neurological convention.

We identified three clusters associated with abstract words processing in a healthy population when only studies reporting abstract visual stimuli were included (Fig. 8), from 4 to 12 different papers (Table 8). Our analyses revealed a robust neural pattern of activity in the frontal and temporal lobes, specifically, the inferior frontal gyrus and the superior and middle temporal gyri.

**Table 8:** Abstract > concrete words—visual stimuli- clusters.

H	Cluster	Macroanatomical location		Cytoarchitectonic label	Weighted Center (MNI; mm)			Vol. (mm^3^)	Peaks: MNI coordinates (mm)			ALE score	Z score	No	Contributors to cluster
		Lobe	Gyrus		x	y	z		x	y	z				Studies
L	1	Frontal, temporal	Inferior frontal, superior temporal	BA 47, BA 45, BA 38	− 50.7	21.7	− 3.5	6992	− 52	24	4	0.029	6.440	12	Perani, 1999 (2); Fiebach, 2004 (1); Sabsevitz, 2005 (3); Binder, 2005 (5); Fliessbach, 2006 (2); Pexman, 2007 (1); Rodríguez-Ferreiro, 2011 (2); Hayashi, 2014 (1); Hoffman, 2015 (3); Skipper, 2014 (2); Wang, 2019 (1); Pauligk, 2019 (2)**
									− 48	20	− 10	0.025	5.900		
									− 54	8	− 18	0.016	4.403		
L	2	Temporal	Middle temporal	BA 22, BA 21	− 60.4	− 44.5	− 1.7	1160	− 60	− 42	− 6	0.016	4.425	5	Noppeney, 2004 (1); Sabsevitz, 2005 (1); Pexman, 2007 (2); Rodríguez-Ferreiro, 2011 (2); Wang, 2019 (1)**
									− 60	− 48	4	0.015	4.273		
L	3	Temporal	Superior temporal	BA 22, BA 21	− 49.5	− 27.5	− 2.6	904	− 50	− 28	− 4	0.016	4.407	4	Sabsevitz, 2005 (1); Hoffman, 2015 (2); Kumar, 2016 (1); Wang, 2019 (1)**

Included 135 stereotactic activation loci from 22 studies, 374 participants, chosen min. cluster size 688 mm^3^.

All the values and labels were extracted from the GingerALE output files. Clusters are ordered for decreasing volume size. Coordinates (x, y, z) are in the MNI space.

H = Hemisphere; ALE = activation likelihood estimation; Nr. = number of studies that contributed to each cluster; L = left; BA = Brodmann area; ** = between brackets are the number of foci from each study that contributed to that specific cluster.

**Figure 9:**
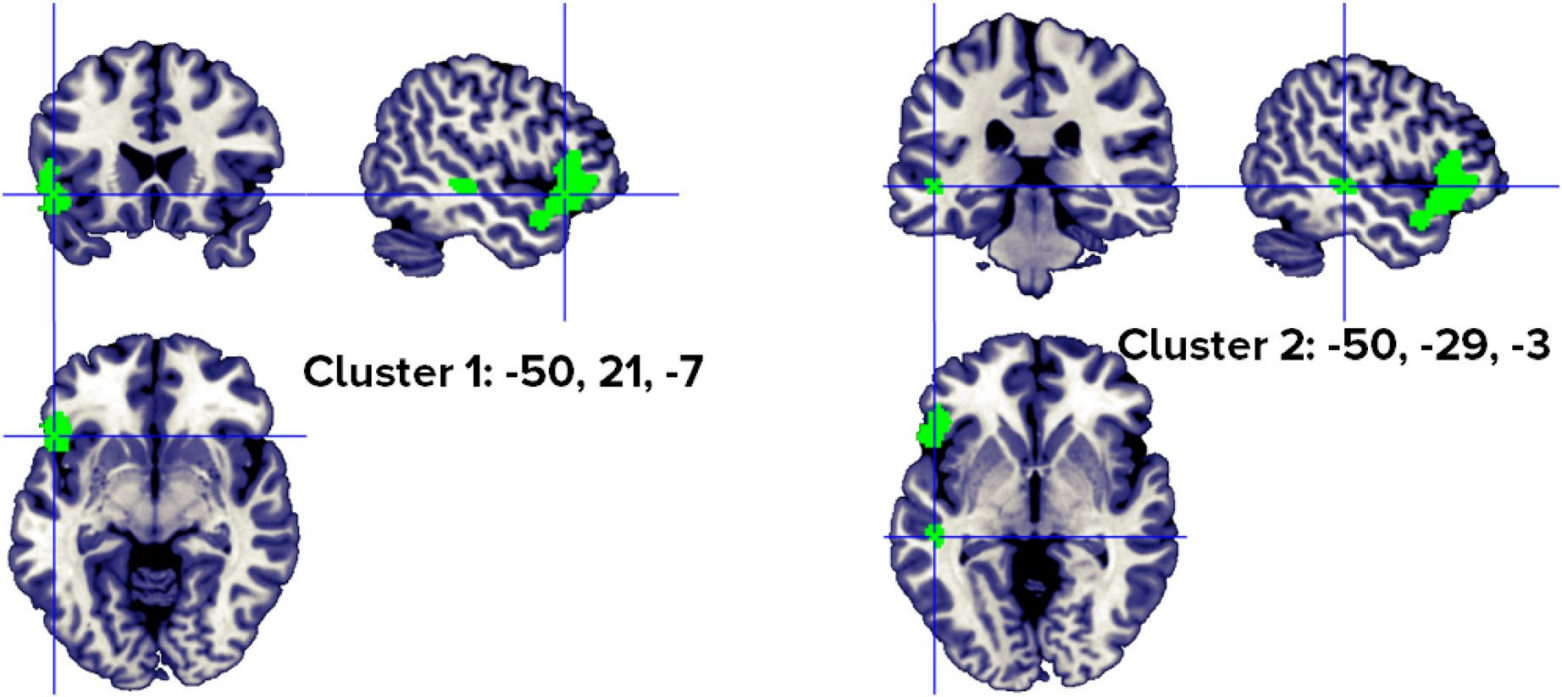
Clusters activated by the abstract > concrete words -semantic and lexical task- contrast. The crosses are centered in the areas correspond to stereotactic coordinates reported in Table 9. The images are presented in neurological convention.

When only foci from lexical and semantic tasks were analyzed, the results indicated 2 clusters (with 1–4 individual peaks each, from 3 to 9 different studies), in the left inferior frontal gyrus, superior and middle temporal gyrus (Fig. 9, Table 9).

**Table 9:** Abstract > concrete words—semantic and lexical task only- clusters.

H	Cluster	Macroanatomical location		Cytoarchitectonic label	Weighted center (MNI; mm)			Vol. (mm^3^)	Peaks: MNI Coordinates (mm)			ALE score	Z Score	Nr	Contributors to cluster
		Lobe	Gyrus		x	y	z		x	Y	z				Studies
L	1	Frontal, temporal	Inferior frontal, Superior temporal	BA 47, BA 38	− 50.3	20.7	− 6.7	6080	− 48	20	− 10	0.025	6.014	9	Perani, 1999 (2); Fiebach, 2004 (1); Sabsevitz, 2005 (3); Binder, 2005 (4); Pexman, 2007 (1); Hoffman, 2015 (3); Skipper, 2014 (2); Wang, 2019 (1); Pauligk, 2019 (2)**
									− 48	30	− 4	0.017	4.737		
									− 54	8	− 18	0.016	4.497		
									− 52	24	6	0.016	4.484		
L	2	Temporal	Superior Temporal Middle Temporal	BA 22, BA 21	− 50	− 29.2	− 2.6	688	− 50	− 28	− 4	0.015	4.340	3	Sabsevitz, 2005 (1); Hoffman, 2015 (2); Wang, 2019 (1)**

Included 116 stereotactic activation loci from 17 studies, 289 participants, Chosen min. cluster size 680 mm^3^

.

All the values and labels were extracted from the GingerALE output files. Clusters are ordered for decreasing volume size. Coordinates (x, y, z) are in the MNI space.

H = Hemisphere; ALE = activation likelihood estimation; Nr. = number of studies that contributed to each cluster; L = left; BA = Brodmann area; ** = between brackets are the number of foci from each study that contributed to that specific cluster.

As previously specified, due to the very small number of studies we could not conduct sub-analyses based on the (1) verbs only, (2) other types of tasks present in the included publications like mental image generation, memory tasks, or perceptual decision task only; (3) auditory stimuli only.

In Table 10 the descriptive information for each sub-analysis is reported.

**Table 10:** Descriptive information for each sub-analysis.

Contrasts	Inclusion criteria:	Participants			Stimuli	Stimuli presentation modality	Experimental task
		Mean sample size	SD	Min–Max			
Words	Imaging technique: PET or fMRI, reported stereotaxic coordinates (in the MNI or Talairach atlases), whole-brain voxel-based data analyses, more than 5 participants in each study, sample population of healthy, adult participants, reported concrete > abstract or abstract > concrete contrast, word stimuli, published in English,						
concrete > abstract words		16	5.2	7/28	concrete and abstract words	visual and auditory	passive listening passive reading perceptual task overt repetition task lexical decision memory encoding task recognition task semantic judgment semantic similarity decision semantic categorization mental image generation
abstract words > concrete words		16.7	5.8	6/28			
Nouns	Imaging technique: PET or fMRI, reported stereotaxic coordinates (in the MNI or Talairach atlases), whole-brain voxel-based data analyses, more than 5 participants in each study, sample population of healthy, adult participants, reported concrete nouns > abstract nouns, or abstract nouns > concrete nouns contrast, nouns stimuli, published in English,						
concrete nouns > abstract nouns		16.7	5.6	7/28	Concrete and abstract nouns	Visual and auditory	Passive listening passive reading perceptual task overt repetition task lexical decision memory encoding task recognition task semantic judgment semantic similarity decision semantic categorization mental image generation
abstract nouns > concrete nouns		18.1	5.7	7/28			
visually presented stimuli	Imaging technique: PET or fMRI, reported stereotaxic coordinates (in the MNI or Talairach atlases), whole-brain voxel-based data analyses, more than 5 participants in each study, sample population of healthy, adult participants, reported concrete > abstract or abstract > concrete contrast, visual presented stimuli, word stimuli, published in English,						
concrete words > abstract words visual stimuli only		16.7	4.9	9/28	Concrete and abstract words	Visual	Passive listening passive reading perceptual task overt repetition task lexical decision memory encoding task recognition task semantic judgment semantic similarity decision semantic categorization mental image generation
abstract words > concrete words visual stimuli only		17.1	5.1	6/28			
lexical and semantic tasks	Imaging technique: PET or fMRI, reported stereotaxic coordinates (in the MNI or Talairach atlases), whole-brain voxel-based data analyses, more than 5 participants in each study, sample population of healthy, adult participants, reported concrete > abstract or abstract > concrete contrast, lexical or semantic decision tasks only, word stimuli, published in English,						
concrete > abstract word (only lexical and semantic tasks)		17.1	5.0	9/28	Concrete and abstract words	Visual and auditory	Lexical decision semantic judgment semantic similarity decision semantic categorization
abstract > concrete word (only lexical and semantic tasks)		17.1	5.6	6/28			

In Appendix A (supplementary materials) we present the analyses without PET data. Except for a few clusters that have a smaller number of voxels and one cluster that fragmented into two smaller ones (for the abstract > concrete contrast), all the other clusters are perfectly overlapped (for detail see from Tables 2A, 3, 4, 5, 6, 7, 8, 9A and from Figs. 1A, 2, 3, 4, 5, 6, 7, 8A). No cluster disappeared and no new clusters were observed, indicating a good data consistency. In Appendix A we also added Table 10. A in which we presented the Brodmann area (BA) for the activated clusters with a brief description.

## Discussion

As we pointed out in the introduction, neuropsychological studies suggest a role of the lateral prefrontal cortex in processing abstract words and of the left anterior temporal lobe in processing concrete ones. These data are not confirmed by neuroimaging studies. We run a meta-analysis using more stringent criteria to assess whether imaging data can support not only this segregation but also in which components the two networks differ. There are many variables that could influence our findings concerning the neural correlates, like the type of task, type of stimuli, stimuli presentation modality. We tried to control for all these factors in order to obtain accurate results. Since the number of studies was limited, we could not analyze data according to type of task (e.g., lexical decision task vs. semantic task), but at least we excluded those studies without a semantic or lexical decision task. The task performed during fMRI scan is particularly relevant because the activation observed during passively hearing/reading words might be very different from the one observed during semantic judgments for concrete vs. abstract (e.g., the decision for—which is better associated to a table: a chair or a bench?). Regarding the stimulus type, there is an ongoing debate concerning nouns vs. verbs in general^38^. This question becomes even more difficult when we try to separate abstract and concrete nouns, and abstract and concrete verbs (we could not analyze verbs separately for the lack of studies). Both, Wang et al.^37^ and Binder et al.^40^ combined different types of stimuli, e.g., words, sentences, fixed expressions such as idioms, and short stories without further focusing on the stimulus type. Furthermore, since Binder et al.’s^40^ objective was to investigate the semantic processing in general and not concrete and abstract distinction (although they run a sub-analysis on these two categories), the activation peaks meta-analyzed were obtained from different contrasts: concrete and abstract stimuli > baseline, concrete > abstract and abstract > concrete stimuli. This choice is comprehensible given their objective but the results could be biased by the type of contrast applied; indeed, discrepancies in the patterns of cortical activation across studies may be attributable, at least in part, to differences in baseline tasks, and hence, reflect the limits of the subtractive logic.

Thirty-two imaging studies were included, which evaluated the activation patterns in response to concrete and abstract concepts. All the data included in the ALEanalysis are based on general linear model, GLM. We also looked for studies that used the more modern multivariate pattern analysis, i.e., a set of methods that analyze neural responses as patterns of activity^90^, in order to have a separate dataset with this type of methods. Unfortunately, we found a very small number of publications preventing a further meta-analytic procedure^48,91,92^.

The results of this meta-analysis, consistent with those of previous ones^37,40^, confirmed that concrete and abstract words processing relies, at least in part, on different brain regions. Based on the currently available data we could not investigate the existence of overlapping networks between concrete and abstract words. The ALE procedure was completely data-driven, without a prior theoretical basis, and the results are constrained only by the nature of our data (e.g., the limited temporal resolution of the neuroimaging techniques, the correlational nature of the data), and by our inclusion/exclusion criteria.

As previously mentioned, experiments testing for greater activation for concrete than abstract words (concrete words > abstract words) converge in the temporo-parieto-occipital regions; namely, the left middle temporal gyrus, left fusiform, left parahippocampal and lingual gyri, bilateral angular gyrus and precuneus, bilateral posterior cingulate, left superior occipital gyrus and left culmen in the cerebellum. The neuroimaging evidence indicates that concrete concept processing is at least partly associated to the perceptual system, and also rely on mental imagery (precuneus, superior occipital gyrus). Binder et al.^40^ found significant overlapping for concrete stimuli in the angular gyrus bilaterally, left mid-fusiform gyrus, left posterior cingulate, and left dorsomedial prefrontal cortex (DMPFC). With the exception of DMPFC that might be related to the stimuli complexity and/or different baselines, all the other regions are confirmed by our data. At variance with Wang et al.’s meta-analysis^37^ we found a bilateral involvement of the posterior cingulate cortex (PCC), angular and precuneus gyri. Although involved in many semantic-based tasks, the function of the PCC in semantic cognition is still debated. The following hypothesis are proposed: (1) this region could act as a supramodal convergence zone^40^, (2) PCC activation could reflect the greater engagement of an imagery-based perceptual system for concrete stimuli, or (3) PCC might be an interface between semantic knowledge and episodic memory^91^. The precuneus also seems associated with visuospatial imagery, a hypothesis supported by experiments conducted on episodic memory retrieval and linguistic tasks which required the processing of high imagery words or mental image generation^83^. The same regions were found when only nouns were considered (concrete nouns > abstract nouns contrast) with the difference that the right hemisphere activation disappeared. The two right hemisphere clusters might be specifically correlated with action verbs but this result could also be a consequence of the lack of power due to the limited number of studies (15 studies in the nouns dataset vs. 22 in the noun-and-verb database).

The results on abstract words replicated those reported by Wang and colleagues^37^ and Binder et al.^40^; higher activation for abstract compared to concrete words conditions (abstract words > concrete words) is more frequently reported in a left lateralized network, encompassing the inferior frontal gyrus (IFG, Brodmann areas 45, 47), a very small portion of the precentral gyrus, the superior and middle temporal gyri, and inferior parietal. They are also in line with the results observed in brain-damaged patients.

It has been suggested that the ventrolateral prefrontal cortex (VLPFC) implements semantic control in two steps^93^. Step 1 constitutes controlled access to stored representations when bottom-up input is not sufficient. Step 2 operates at post-retrieval and is thought to bias competition among representations that have been activated during Step 1. According to Badre and Wagner^94^, both steps recruit VLPFC, though different parts of it, with BA 45 involved in Step 2. In other words, IFG activation could reflect a higher level of semantic control processes (additional resources) since abstract stimuli might require semantic selection, irrelevant cues inhibition, effortful integration, top-down control and working-memory related processes^95^, in agreement with the context availability theory^96^. In line with this hypothesis, this region showed greater activation for abstract words when a judgment task was performed following irrelevant cues and reduced activation when semantic decisions were made with contextual help, supporting the idea that this area responds more strongly to abstract words because their meanings are inherently more variable and require more control during linguistic processing as compared to the concrete ones^53,97^. An alternative explanation is offered by Della Rosa^98^ using a lexical decision task; they found that the left IFG was particularly active during presentation of words characterized by low imageability and low context availability. The authors’ interpretation was that this area could be a functional convergence zone between imageability and context availability, differentiating abstract from concrete concepts.

In neuroimaging studies, besides the IFG, additional clusters were found in the left superior and middle temporal lobe. However, when only nouns were considered (and not verbs), these clusters lost significance, supporting the idea that the cerebral networks deputed to noun and verb processing might be slightly different.

On the other side, results on concrete words do not support neuropsychological data. Indeed, apart from several single case reports, a study comparing the behavioral variant of frontotemporal dementia (FTD), in which there is a predominant prefrontal atrophy, to the semantic variant, with anterior temporal atrophy showed that while the former group had an increase of the concreteness effect, the reversal was found in the semantic variant group. Similarly, patients with left Anterior Temporal Lobe (ATL) resection show the same pattern of reversal concreteness effect^33^.

One possibility of this inconsistent results is the type of task; the neuroimaging studies used pleasantness judgments, memory tasks, lexical decision, etc. while, in general, patients are examined by means of naming and comprehension tasks and, occasionally, also semantic judgments. Orena et al.^36^, for example, using direct electrical stimulation (DES) for brain mapping during awake surgery found no behavioral differences between BA 44 and BA 38 stimulation while patients performed a lexical decision task, but they registered a dissociation between abstract and concrete words during a concreteness judgment task; in particular, abstracts words were impaired during stimulation of BA 44 and concrete words during BA 38 stimulation.

Neuroimaging studies are often hard to compare, and many variables could influence the reported results as the duration of the stimuli presentation, stimuli number, stimulus types. For example, in the same type of experiment a large number of stimuli [e.g., 164 nouns^74^] were presented while in other studies, only four words were repeated for more than 140 trials^78^. Moreover, selected stimuli greatly varied among studies encompassing emotions, mind states, living and nonliving things, of different frequency of use, age of acquisition and imageability. In addition, many studies used interchangeably “concreteness” and “imageability” , which are in fact two distinct properties that can differently affect naming and recall^99–101^.

We also controlled for presentation modality. When only visually presented words were included in the analysis no relevant differences were observed between auditory and visual stimuli combined, and only visually presented words (see Figs. 5, 9). This can be partially due to the very small number of studies using auditory information (only 5 studies out of 32).

Another relevant element is the participants’ age since aging can modify neural organization due to neuroplasticity^102^. With two exceptions^69,77^ in which the participants’ mean age was > 70, all other studies included a young population with a mean age < 30 (see Table 1). Neuropsychological studies (on patients) involve a different population ranging from 55 to 75. Information obtained from healthy young people cannot be optimal to interpret data from elderly, brain-damaged patients.

According to Eickhoff et al.^46^, the statistical power of the current meta-analysis to detect not only large, but also small- and medium-size effects can be considered acceptable. Nevertheless, meta-analytic power is intrinsically limited by the number of currently available data especially for two sub-analyses: (1) concrete nouns > abstract nouns, only 15 independent experiments, and (2) lexical and semantical task—concrete words > abstract words, 16 studies. This indicates that, in these two cases, we cannot properly control the influence of individual experiments and that we might have failed to detect small effects. Another limitation is related to the sample size of the included experiments that ranged from 6 to 28 participants. We acknowledge the need to consider only well-designed and controlled studies but taking into account the limited number of papers we were forced to include data from studies with uncorrected *p* values (see Table 1) risking subtle activation differences that may underlie abstract-concrete differences.

This meta-analysis is focused on how representations of abstract and concrete words are processed in the brain. Regarding this last point, future research should better understand the specific role of each region within the semantic network, how they are connected, and specify how task and stimuli characteristics interact and modify activation patterns.

Considering the main question, we can confirm that concrete and abstract words involve at least partially segregated brain areas, the IFG being relevant for abstract nouns and verbs; in contrast, we could not find evidence of the ATL involvement for concrete items. Our data indicate a more posterior activation for concrete words in regions that are often correlated with mental imagery processes. This meta-analysis seems to support the hypothesis that abstract and concrete words have partly separate neural correlates but the specific features that differentiate between these two classes of stimuli are still open to discussion. The cortical regions that are commonly activated in imaging studies investigating concrete and abstract words seem more congruent with the Dual Coding Theory^10^, i.e., concrete words have richer representations, depending on both hemispheres. Regarding the hub-and-spoke model (hub regions interacting with modality-specific processing areas), we observed activation patterns in areas considered neural crossroads of the semantic network like the posterior cingulate region, the anterior temporal lobe, and the left inferior frontal gyrus^91,98,103^ , but these data cannot be interpreted in the frame of this theoretical model.

The lack of converging evidence from clinical neuropsychological and neuroimaging data might be explained by several variables like task and stimuli type, differences in terms of age and brain plasticity between the two populations (young vs. elderly people), etc. These discrepancies deserve further investigation, for example by means of balanced groups of healthy and clinical participants, combining different techniques in the same experiment as TMS-EEG, or TMS and fMRI.

## Supplementary Information

Supplementary Information.
