# Supplementary material for: An ALE meta-analytical review of the neural correlates of abstract and concrete words
Source: Sci Rep. 2021 Aug 3;11:15727. doi: 10.1038/s41598-021-94506-9 (PMC8333331; doi:10.1038/s41598-021-94506-9)
Supplement: Supplementary file 1 — Supplementary Information. [file 41598_2021_94506_MOESM1_ESM.docx]

**An ALE meta-analytical review of the neural correlates of Abstract and Concrete Words.**

**Appendix A (supplementary materials)**

Madalina Bucur^a^ and Costanza Papagno ^a,b*^

^a^ Center for Mind/Brain Sciences (CIMeC), University of Trento, Italy

^b^ Department of Psychology, University of Milano-Bicocca

* Corresponding author:

Costanza Papagno, MD, PhD

CeRiN (Center for Cognitive Neurorehabilitation)

CIMeC, University of Trento

Via Matteo del Ben 5/b

38068 Rovereto (TN)

Italy

e-mail: costanza.papagno@unitn.it

tel 0464 808165

**Materials and methods**

After excluding the four PET studies, Mellet, (1998), Perani, (1999), Tyler, (2001), Whatmough, (2004), the meta-analysis is based on 28 fMRI studies exploring the neural basis of concrete and abstract words representation (for details see Table 1.A).

| **Table 1.A \| Descriptive information of the 32 experiments included in the meta-analysis.** | | | | | | | | | | |
| --- | --- | --- | --- | --- | --- | --- | --- | --- | --- | --- |
|  | Paper | Technique | Sample size | Age of subjects (years) | Stimuli | Stimuli presentation modality | Experimental task | Design | Random or fixed effect | Contrasts  p-value * |
| 1. | D'Esposito, M., Detre, J. A., Aguirre, G. K., Stallcup, M., Alsop, D. C., Tippet, L. J., & Farah, M. J. (1997). A functional MRI study of mental image generation. Neuropsychologia, 35(5), 725-730. | MRI, 1.5 Tesla | 7 | range  18–37 | English nouns  (concrete vs abstract) | Auditory | mental image generation (concrete) and passive listening (abstract) | blocks | fixed | p < .001 corrected  voxel-wise |
|  | ~~Mellet, E., Tzourio, N., Denis, M., & Mazoyer, B. (1998). Cortical anatomy of mental imagery of concrete nouns based on their dictionary definition. Neuroreport, 9(5), 803-808.~~ | ~~PET~~ | ~~8~~ | ~~range~~  ~~20–25~~ | ~~French nouns~~  ~~(concrete vs abstract)~~ | ~~Auditory~~ | ~~mental image generation (concrete) and passive listening (abstract)~~ | ~~blocks~~ | ~~fixed~~ | ~~p = 0.001 uncorrected~~ |
|  | ~~Perani, D., Cappa, S. F., Schnur, T., Tettamanti, M., Collina, S., Rosa, M. M., & Fazio1, F. (1999). The neural correlates of verb and noun processing: A PET study. Brain, 122(12), 2337-2344.~~ | ~~PET~~ | ~~14~~ | ~~range~~  ~~22–26~~ | ~~Italian words:~~  ~~(i) concrete verbs,~~  ~~(ii) abstract verbs,~~  ~~(iii) concrete nouns,~~  ~~(iv) abstract nouns~~ | ~~Visual~~ | ~~lexical decision (classify stimuli as words or nonwords)~~ | ~~blocks~~ | ~~fixed~~ | ~~p < 0.001 uncorrected~~ |
| 2. | Kiehl, K. A., Liddle, P. F., Smith, A. M., Mendrek, A., Forster and, B. B., & Hare, R. D. (1999). Neural pathways involved in the processing of concrete and abstract words. Human brain mapping, 7(4), 225-233. | MRI, 1.5 Tesla | 6 | range  22–26 | English words:  concrete or abstract | Visual | lexical decision (classify stimuli as words or nonwords) | blocks | fixed | p < 0.05 corrected  voxel-wise |
| 3. | Jessen, F., Heun, R., Erb, M., Granath, D. O., Klose, U., Papassotiropoulos, A., & Grodd, W. (2000). The concreteness effect: Evidence for dual coding and context availability. Brain and language, 74(1), 103-112. | MRI, 1.5 Tesla | 14 | range  20–44  (31.5 ± 6.3) | German nouns:  concrete or abstract | Visual | memory encoding task | blocks | fixed | p < 0.001 uncorrected |
|  | ~~Tyler, L. K., Russell, R., Fadili, J., & Moss, H. E. (2001). The neural representation of nouns and verbs: PET studies. Brain, 124(8), 1619-1634.~~ | ~~PET~~ | ~~9~~ | ~~range~~  ~~21–34~~  ~~26 ± 5~~ | ~~English words:~~  ~~concrete or abstract~~ | ~~Visual~~ | ~~lexical decision (classify stimuli as words or nonwords)~~ | ~~block~~ | ~~fixed-effect~~ | ~~p < 0.05 corrected~~  ~~voxel-wise~~ |
| 4. | Grossman, M., Koenig, P., DeVita, C., Glosser, G., Alsop, D., Detre, J., & Gee, J. (2002). The neural basis for category-specific knowledge: an fMRI study. Neuroimage, 15(4), 936-948. | MRI, 1.5 Tesla | 16 | mean age  23.4 | English nouns:  animals, implement, abstract | Visual | semantic judgment (pleasant or not) | blocks | fixed | p < 0.05 corrected  voxel-wise |
| 5. | Kounios, J., Koenig, P., Glosser, G., DeVita, C., Dennis, K., Moore, P., & Grossman, M. (2003). Category-specific medial temporal lobe activation and the consolidation of semantic memory: evidence from fMRI. Cognitive brain research, 17(2), 484-494. | MRI, 1.5 Tesla | 16 | mean age  73.9 | English nouns:  animal, implement,  and abstract | visual | semantic judgement  (pleasant or not) | block | fixed-effect | p < 0.05 corrected |
|  | ~~Whatmough, C., Verret, L., Fung, D., & Chertkow, H. (2004). Common and contrasting areas of activation for abstract and concrete concepts: An H215O PET study. Journal of Cognitive Neuroscience, 16(7), 1211-1226.~~ | ~~PET~~ | ~~15~~ | ~~range~~  ~~69-90~~  ~~74.3 ± 5.6~~ | ~~English nouns:~~  ~~two pairs (concrete or abstract)~~ | ~~Visual~~ | ~~semantic similarity decision (read aloud if the pairs are similar in meanings)~~ | ~~ns~~ | ~~ns~~ | ~~p < 0.05 corrected~~  ~~voxel-wise~~ |
| 6. | Noppeney, U., & Price, C. J. (2004). Retrieval of abstract semantics. Neuroimage, 22(1), 164-170. | MRI, 2 Tesla | 15 | range  21–46  mean age 30 | English  (i) abstract concepts,  (ii) hand movements,  (iii) visual attributes  (iv) sounds | Visual | semantic similarity decision | blocks | random | p < 0.001 uncorrected |
| 7. | Fiebach, C. J., & Friederici, A. D. (2004). Processing concrete words: fMRI evidence against a specific right-hemisphere involvement. Neuropsychologia, 42(1), 62-70. | MRI, 3 Tesla | 12 | mean age 25 | German nouns:  abstract and concrete | Visual | lexical decision (classify stimuli as words or nonwords) | event-related | ns | p < 0.05 corrected  cluster-wise |
| 8. | Giesbrecht, B., Camblin, C. C., & Swaab, T. Y. (2004). Separable effects of semantic priming and imageability on word processing in human cortex. Cerebral Cortex, 14(5), 521-529. | MRI, 1.5 Tesla | 10 | ns | English words:  high imageable and low imageable | Visual | semantic judgement (words pairs related or unrelated) | event-related | random | P < 0.005 uncorrected |
| 9. | Sabsevitz, D. S., Medler, D. A., Seidenberg, M., & Binder, J. R. (2005). Modulation of the semantic system by word imageability. Neuroimage, 27(1), 188-200. | MRI, 1.5 Tesla | 28 | range  18–33  22.8 ± 3.6 | English nouns:  concrete and abstract triads | Visual | semantic similarity decision | event-related | random | p < .001, uncorrected |
| 10. | Binder, J. R., Westbury, C. F., McKiernan, K. A., Possing, E. T., & Medler, D. A. (2005). Distinct brain systems for processing concrete and abstract concepts. Journal of cognitive neuroscience, 17(6), 905-917. | MRI, 1.5 Tesla | 24 | range  20–50 | English nouns:  abstract and concrete | Visual | lexical decision (classify stimuli as words or nonwords) | event-related | random | p < .005 uncorrected |
| 11. | Harris, G. J., Chabris, C. F., Clark, J., Urban, T., Aharon, I., Steele, S., ... & Tager-Flusberg, H. (2006). Brain activation during semantic processing in autism spectrum disorders via functional magnetic resonance imaging. Brain and cognition, 61(1), 54-68. | MRI, 1.5 Tesla | 20 | range  19–50  31 ± 9 | English nouns:  abstract and concrete | Visual | semantic judgment (positive or negative) | block | random | p < 0.05 corrected  cluster-wise |
| 12. | Fliessbach, K., Weis, S., Klaver, P., Elger, C. E., & Weber, B. (2006). The effect of word concreteness on recognition memory. NeuroImage, 32(3), 1413-1421. | MRI, 1.5 Tesla | 21 | range  19–43  27.4 ± 6.2 | German nouns:  abstract and concrete | Visual | recognition task (old/new-decision) | event-related | random | p < 0.05 corrected  cluster-wise |
| 13. | Rüschemeyer, S. A., Brass, M., & Friederici, A. D. (2007). Comprehending prehending: neural correlates of processing verbs with motor stems. Journal of cognitive neuroscience, 19(5), 855-865. | MRI, 3 Tesla | 20 | range  22–33  27 ±3 | German verbs: simple, complex, motor, abstract | visual | lexical decision (classify stimuli as words or nonwords) | block | random | p < .001, uncorrected |
| 14. | Pexman, P. M., Hargreaves, I. S., Edwards, J. D., Henry, L. C., & Goodyear, B. G. (2007). Neural correlates of concreteness in semantic categorization. Journal of Cognitive Neuroscience, 19(8), 1407-1419. | MRI, 3 Tesla | 20 | 26.5 ± 4.5 | English nouns:  abstract and concrete | Visual | semantic categorization (consumable or not) | event-related | random | p < 0.05  ns |
| 15. | Van Dam, W. O., Rueschemeyer, S. A., & Bekkering, H. (2010). How specifically are action verbs represented in the neural motor system: an fMRI study. Neuroimage, 53(4), 1318-1325. | MRI, 3 Tesla | 16 | range  18–38  24 ± 4.63 | Dutch verbs denoting  (i) actions that you perform mostly with your arms/hands/ mouth or  (ii) abstract events | Visual | semantic categorization task (go – no go) | event-related | random | p < 0.05 corrected |
| 16. | Zhuang, J., Randall, B., Stamatakis, E. A., Marslen-Wilson, W. D., & Tyler, L. K. (2011). The interaction of lexical semantics and cohort competition in spoken word recognition: an fMRI study. Journal of Cognitive Neuroscience, 23(12), 3778-3790. | MRI, 3 Tesla | 14 | range  19–33 | British English nouns manipulating (cohort competition and imageability) | Auditory | lexical decision (classify stimuli as words or nonwords) | event-related | random | p < 0.05 corrected  cluster-wise |
| 17. | Rodríguez-Ferreiro, J., Gennari, S. P., Davies, R., & Cuetos, F. (2011). Neural correlates of abstract verb processing. Journal of Cognitive Neuroscience, 23(1), 106-118. | MRI, 3 Tesla | 14 | range  23–35  mean 29 | Spanish verbs:  concrete and abstract | Visual | passive reading | block | mixed effects | p < .001, uncorrected |
| 18. | van Dam, W. O., van Dijk, M., Bekkering, H., & Rueschemeyer, S. A. (2012). Flexibility in embodied lexical‐semantic representations. Human brain mapping, 33(10), 2322-2333. | MRI, 3 Tesla | 20 | range  18–24  20.5 ± 2.2 | Dutch  (1) action color  (2) action nouns  (3) color  (4) abstract nouns | Auditory | semantic categorization (action or color characteristics) | block | random | p < 0.005  ns |
| 19. | Wilson-Mendenhall, C. D., Simmons, W. K., Martin, A., & Barsalou, L. W. (2013). Contextual processing of abstract concepts reveals neural representations of nonlinguistic semantic content. Journal of cognitive neuroscience, 25(6), 920-935. | MRI, 3 Tesla | 13 | range  18-24 | English words:  two abstract (convince, arithmetic)  two concrete (rolling, red) | Visual | semantic task (concept–scene match) | block | random | p<0.05  corrected  voxel-wise |
| 20. | Vigliocco, G., Kousta, S. T., Della Rosa, P. A., Vinson, D. P., Tettamanti, M., Devlin, J. T., & Cappa, S. F. (2013). The neural representation of abstract words: the role of emotion. Cerebral Cortex, 24(7), 1767-1777. | MRI, 3 Tesla | 20 | range  18–33  21.9 ± 4.4 | English nouns:  abstract and concrete | Visual | lexical decision (classify stimuli as words or nonwords) | block | random | P < 0.05 FWE-cluster-wise |
| 21. | Hayashi, A., Okamoto, Y., Yoshimura, S., Yoshino, A., Toki, S., Yamashita, H., ... & Yamawaki, S. (2014). Visual imagery while reading concrete and abstract Japanese kanji words: An fMRI study. *Neuroscience research*, *79*, 61-66. | MRI, 1.5 Tesla | 16 | range  20-36  26.1 ± 5.9 | Japanese kanji nouns:  concrete and abstract | Visual | generate visual imagery | block | random | p < .001, uncorrected |
| 22. | Roxbury, T., McMahon, K., & Copland, D. A. (2014). An fMRI study of concreteness effects in spoken word recognition. Behavioral and Brain Functions, 10(1), 34. | MRI, 3 Tesla | 17 | 27 ± 5.1 | English nouns: concrete, abstract and pseudowords | auditory | lexical decision (classify stimuli as words or nonwords) | event-related | random | p < .001, uncorrected |
| 23. | Skipper, L. M., & Olson, I. R. (2014). Semantic memory: Distinct neural representations for abstractness and valence. Brain and Language, 130, 1-10. | MRI, 3 Tesla | 19 | mean age 23 | English nouns:  concrete and abstract | Visual | semantic task (answer to question in reference to the 3 words in the block) | block | ns | p<0.001 FDR  corrected  cluster-wise |
| 24. | Hoffman, P., Binney, R. J., & Ralph, M. A. L. (2015). Differing contributions of inferior prefrontal and anterior temporal cortex to concrete and abstract conceptual knowledge. *Cortex*, *63*, 250-266. | MRI, 3 Tesla | 20 | range  20-39  mean: 25 | English words:  concrete and abstract | Visual | semantic task (synonym judgement) | block | random | p<0.05 corrected  cluster-wise |
| 25. | Kumar, U. (2016). Neural dichotomy of word concreteness: a view from functional neuroimaging. *Cognitive processing*, *17*(1), 39-48. | MRI, 3 Tesla | 20 | 28.3 ± 3. | Hindi nouns: abstract, concrete and non-words | Visual | perceptual task (orthography judgment) | block | fixed | p<0.05 corrected |
| 26 | Wang, X., Wang, B., & Bi, Y. (2019). Close yet independent: Dissociation of social from valence and abstract semantic dimensions in the left anterior temporal lobe. Human brain mapping, 40(16), 4759-4776. | MRI, 3 Tesla | 23 | range  19-29  mean 22.17 | Chinese nouns: abstract, concrete | Visual | semantic task (which of the choices was more semantically related to the probe) | block | ns | p < 0.05 FWE  corrected  cluster-level |
| 27 | Pauligk, S., Kotz, S. A., & Kanske, P. (2019). Differential impact of emotion on semantic processing of abstract and concrete words: ERP and fMRI evidence. Scientific reports, 9(1), 1-13. | MRI, 3 Tesla | 21 | 23.3± 1.9 | German nouns:  abstract and concrete | Visual | delayed lexical decision task  (classify stimuli as words or nonwords) | block | ns | p = 0.001  corrected  voxel-wise |
| 28 | Meersmans, K., Bruffaerts, R., Jamoulle, T., Liuzzi, A. G., De Deyne, S., Storms, G., ... & Vandenberghe, R. (2020). Representation of associative and affective semantic similarity of abstract words in the lateral temporal perisylvian language regions. NeuroImage, 217, 116892. | MRI, 3 Tesla | 26 | range  18-34  22.9± 3.7 | Dutch nouns:  abstract and concrete | visual and  auditory | overt repetition task | event-related | random | p < 0.001  uncorrected  p < 0.05  FWE-corrected |

Abbreviations: ns, not specified

Age is reported in years and when was specified the means and standard deviations are presented

Note: The p values (the statistical threshold for the neuroimaging univariate analysis conducted in the included papers) are reported as they were presented in the original articles; the exact value and the correction procedure was not always specified.

**Results**

***CONCRETE > ABSTRACT Meta-analysis***

**Fig. 1.A** Clusters activated by the **concrete > abstract words contrast**.

The crosses are centered in the areas correspond to stereotactic coordinates reported in Table 2.A.

The images are presented in neurological convention.

In red the clusters from PET and fMRI data combined, overlaid in green the clusters from fMRI data only.

***
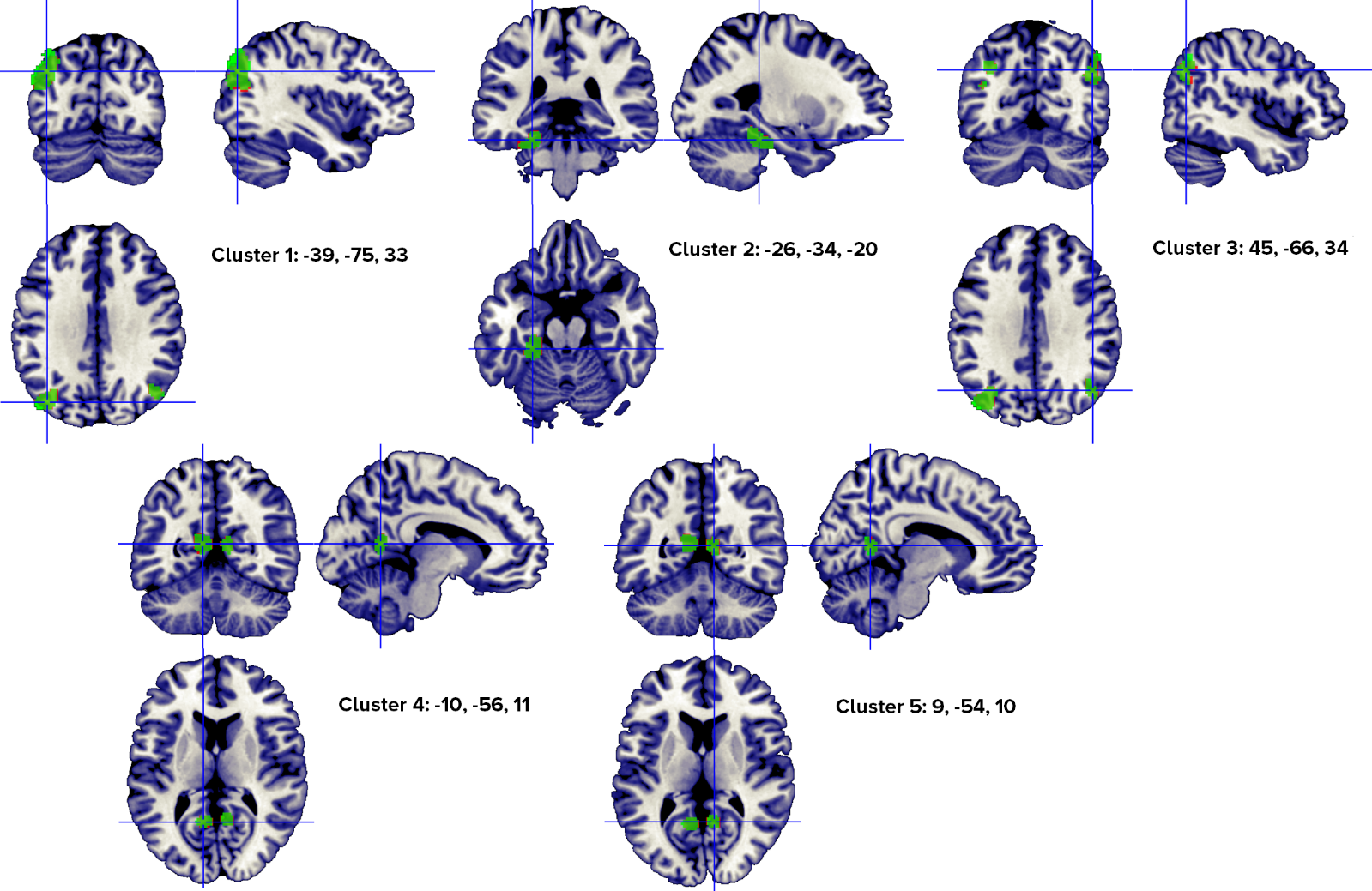
***

| **Table 2.A \|Concrete > Abstract Word Clusters, fMRI data**  included 138 stereotactic activation loci from 19 studies, 321 participants  Chosen min. cluster size 696 mm^3^ | | | | | | | | | | | | | | |
| --- | --- | --- | --- | --- | --- | --- | --- | --- | --- | --- | --- | --- | --- | --- |
| **H** | **Cluster** | **Macroanatomical Location** | | **Cytoarchitectonic Label** | **Weighted Center (MNI; mm)** | | | **Vol. (mm^3^)** | **Peaks: MNI Coordinates (mm)** | | | **ALE score** | **Contributors to cluster** | |
|  |  | **Lobe** | **Gyrus** |  | **x** | **y** | **z** |  | **x** | **y** | **z** |  | **Nr.** | **Studies** |
| L | 1 | Temporal  Occipital  Parietal | Precuneus  Superior Occipital  Middle Temporal  Angular Gyrus | BA 39, BA 19 | -39 | -75 | 33 | 5600 | -40 | -74 | 34 | 0,025 | 12 | Jessen, 2000 (1); Sabsevitz, 2005 (3); Binder, 2005 (1); Harris, 2006 (1); van Dam, 2010 (1); Fliessbach, 2006 (1) Zhuang, 2011 (1); Rodríguez-Ferreiro, 2011 (2); van Dam, 2012 (1); Roxbury, 2014 (1); Skipper, 2014 (5); Hoffman, 2015 (2) ** |
|  |  |  |  |  |  |  |  |  | -44 | -78 | 24 | 0,020 |  |  |
|  |  |  |  |  |  |  |  |  | -38 | -74 | 46 | 0,016 |  |  |
|  |  |  |  |  |  |  |  |  | -40 | -70 | 22 | 0,015 |  |  |
|  |  |  |  |  |  |  |  |  |  |  |  |  |  |  |
| L | 2 | Cerebellum Anterior Lobe  Limbic Lobe  Temporal | Culmen (cerebellum)  Parahippocampal  Fusiform | BA 35, BA 36 | -26 | -34 | -20 | 2560 | -24 | -36 | -18 | 0,021 | 7 | Sabsevitz, 2005 (2); Harris, 2006 (1); Rodríguez-Ferreiro, 2011 (2); van Dam, 2012 (1); Hayashi, 2014 (1); Roxbury, 2014 (1); Hoffman, 2015 (2) ** |
|  |  |  |  |  |  |  |  |  | -24 | -30 | -22 | 0,019 |  |  |
|  |  |  |  |  |  |  |  |  | -34 | -36 | -24 | 0,013 |  |  |
|  |  |  |  |  |  |  |  |  |  |  |  |  |  |  |
| R | 3 | Parietal  Temporal | Inferior Parietal  Angular  Precuneus,  Middle Temporal | BA 39 | 45 | -66 | 34 | 1784 | 44 | -68 | 32 | 0,017 | 5 | Sabsevitz, 2005 (2); van Dam, 2010 (1); Rüschemeyer, 2007 (1); Roxbury, 2014 (1); Hoffman, 2015 (2) ** |
|  |  |  |  |  |  |  |  |  | 42 | -60 | 36 | 0,012 |  |  |
|  |  |  |  |  |  |  |  |  | 48 | -66 | 44 | 0,012 |  |  |
|  |  |  |  |  |  |  |  |  |  |  |  |  |  |  |
| L | 4 | Limbic  Occipital | Posterior Cingulate  Lingual Gyrus  Cuneus | BA 30, BA 18 | -10 | -56 | 11 | 1192 | -10 | -56 | 12 | 0,018 | 5 | Sabsevitz, 2005 (1); Binder, 2005(1); Harris, 2006(1); Rüschemeyer, 2007 (1); Roxbury, 2014(1) ** |
|  |  |  |  |  |  |  |  |  |  |  |  |  |  |  |
| R | 5 | Limbic Lobe | Posterior Cingulate | BA 30 | 9 | -54 | 10 | 840 | 8 | -54 | 10 | 0,019 | 4 | Sabsevitz, 2005 (1); Harris, 2006 (1); Rodríguez-Ferreiro, 2011 (1); Hoffman, 2015 (1) ** |

**Note:** All the values and labels were extracted from the GingerALE output files. Clusters are ordered for decreasing volume size. Coordinates (x, y, z) are in the MNI space.

**Abbreviations:** H=Hemisphere; ALE = activation likelihood estimation; Nr. = number of studies that contributed to each cluster; L = left; BA = Brodmann area; ** = between brackets are the number of foci from each study that contributed to that specific cluster; R = right.

**Fig. 2.A** Clusters activated by the **concrete > abstract nouns contrast**, fMRI data.

The crosses are centered in the areas correspond to stereotactic coordinates reported in Table 3.A

The images are presented in neurological convention.

In red the clusters from PET and fMRI data combined, overlaid in green the clusters from fMRI data only.


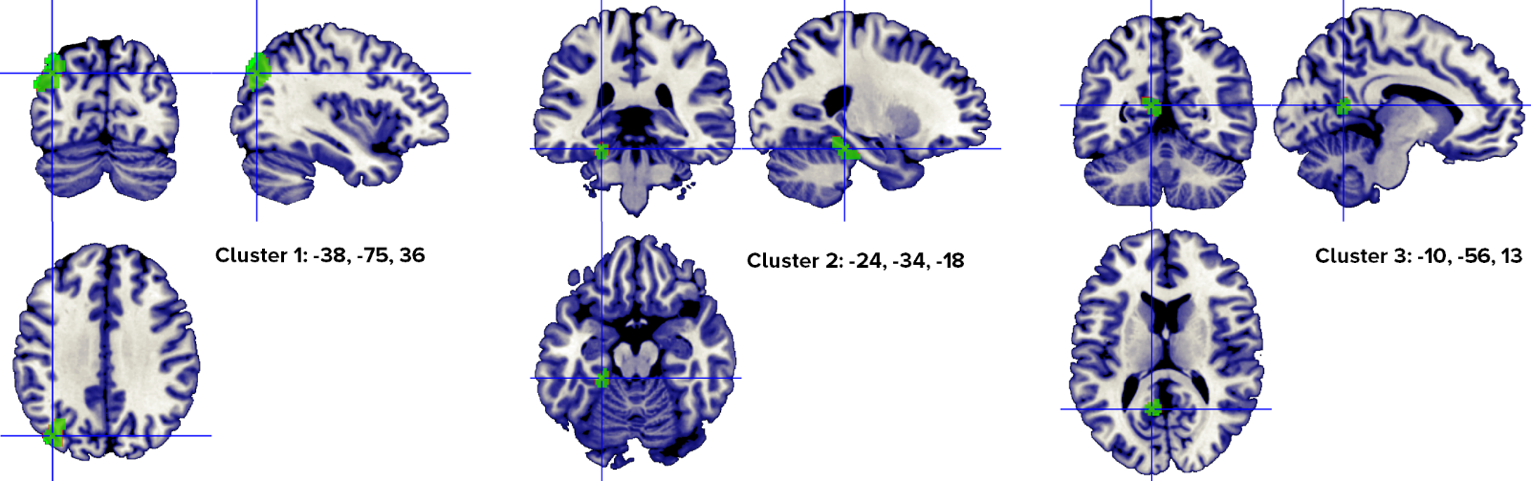


| **Table 3.A \|Concrete > Abstract Nouns Clusters, fMRI data**  included 97 stereotactic activation loci from 13 studies, 228 participants  Chosen min. cluster size 592 mm^3^ | | | | | | | | | | | | | | |
| --- | --- | --- | --- | --- | --- | --- | --- | --- | --- | --- | --- | --- | --- | --- |
| **H** | **Cluster** | **Macroanatomical Location** | | **Cytoarchitectonic Label** | **Weighted Center**  **(MNI; mm)** | | | **Vol. (mm3)** | **Peaks: MNI Coordinates (mm)** | | | **ALE score** | **Contributors to cluster** | |
|  |  | **Lobe** | **Gyrus** |  | **x** | **y** | **z** |  | **x** | **y** | **z** |  | **No.** | **Studies** |
| L | 1 | Occipital  Parietal  Temporal | Superior Occipital,  Precuneus,  Middle Temporal, Angular | BA 39, BA 19 | -38 | -75 | 36 | 4192 | -34 | -68 | 36 | 0.019 | 9 | Jessen, 2000 (1); Sabsevitz, 2005 (3); Binder, 2005 (1); Harris, 2006 (1); Fliessbach (1); Zhuang, 2011 (1); van Dam, 2012 (1); Roxbury, 2014 (1); Skipper, 2014 (4) ** |
|  |  |  |  |  |  |  |  |  | -38 | -74 | 32 | 0.019 |  |  |
|  |  |  |  |  |  |  |  |  | -34 | -78 | 38 | 0.017 |  |  |
|  |  |  |  |  |  |  |  |  | -38 | -74 | 46 | 0.016 |  |  |
|  |  |  |  |  |  |  |  |  | -46 | -76 | 28 | 0.015 |  |  |
|  |  |  |  |  |  |  |  |  |  |  |  |  |  |  |
| L | 2 | Anterior  Limbic | Culmen, Parahippocampal | BA 36, BA 35 | -24 | -34 | -18 | 1432 | -24 | -36 | -18 | 0.015 | 4 | Hayashi, 2004 (1); Sabsevitz, 2005 (2); Harris, 2006 (2); Roxbury, 2014 (1) ** |
|  |  |  |  |  |  |  |  |  |  |  |  |  |  |  |
| L | 3 | Limbic  Occipital | Posterior Cingulate, Cuneus | BA 30, BA 29 | -10 | -56 | 13 | 1040 | -10 | -56 | 12 | 0.017 | 4 | Sabsevitz, 2005 (1); Binder, 2005 (2); Harris, 2006 (1); Roxbury, 2014 (1) ** |
|  |  |  |  |  |  |  |  |  | -8 | -46 | 14 | 0.009 |  |  |

**Note:** All the values and labels were extracted from the GingerALE output files. Clusters are ordered for decreasing volume size. Coordinates (x, y, z) are in the MNI space.

**Abbreviations:** H=Hemisphere; ALE = activation likelihood estimation; Nr. = number of studies that contributed to each cluster; L = left; BA = Brodmann area; ** = between brackets are the number of foci from each study that contributed to that specific cluster

**Fig. 3.A** Clusters activated by the **concrete > abstract words - visual stimuli – contrast**, fMRI data.

The crosses are centered in the areas correspond to stereotactic coordinates reported in Table 4.A

The images are presented in neurological convention.

In red the clusters from PET and fMRI data combined, overlaid in green the clusters from fMRI data only.


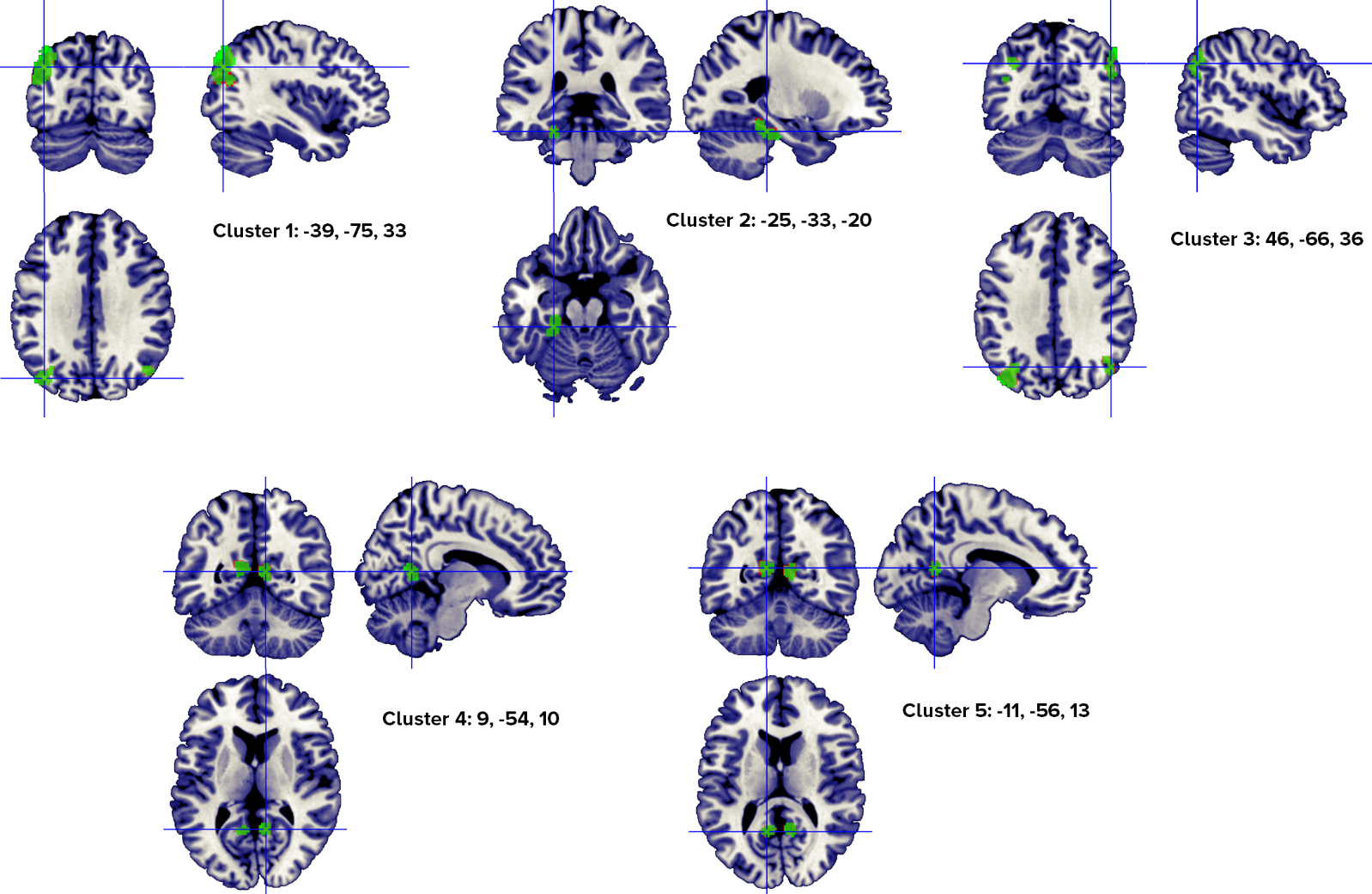


| **Table 4.A \|Concrete > Abstract Words - Visual stimuli- Clusters, fMRI data**  included 119 stereotactic activation loci from 16 studies, 277 participants  Chosen min. cluster size 712 mm^3^ | | | | | | | | | | | | | | |
| --- | --- | --- | --- | --- | --- | --- | --- | --- | --- | --- | --- | --- | --- | --- |
| **H** | **Cluster** | **Macroanatomical Location** | | **Cytoarchitectonic Label** | **Weighted Center (MNI; mm)** | | | **Vol. (mm3)** | **Peaks: MNI Coordinates (mm)** | | | **ALE score** | **Contributors to cluster** | |
|  |  | **Lobe** | **Gyrus** |  | **x** | **y** | **z** |  | **x** | **y** | **z** |  | **No.** | **Studies** |
| L | 1 | Temporal  Occipital  Parietal | Superior Occipital  Middle Temporal  Precuneus  Angular | BA 19, BA 39 | -39 | -75 | 33 | 5120 | -40 | -76 | 34 | 0.021 | 10 | Jessen, 2000 (1); Sabsevitz, 2005 (1); Binder, 2005 (3); Harris, 2006 (1); Fliessbach, 2006 (1); van Dam, 2010 (1); Zhuang, 2011 (1); Rodríguez-Ferreiro, 2011 (2); Skipper, 2014 (5); Hoffman, 2015 (2) ** |
|  |  |  |  |  |  |  |  |  | -44 | -78 | 24 | 0.017 |  |  |
|  |  |  |  |  |  |  |  |  | -36 | -78 | 38 | 0.017 |  |  |
|  |  |  |  |  |  |  |  |  | -34 | -68 | 36 | 0.017 |  |  |
|  |  |  |  |  |  |  |  |  | -38 | -74 | 46 | 0.016 |  |  |
|  |  |  |  |  |  |  |  |  | -40 | -70 | 22 | 0.014 |  |  |
|  |  |  |  |  |  |  |  |  |  |  |  |  |  |  |
| L | 2 | Limbic Lobe, Anterior,  Temporal | Parahippocampal  Culmen  Fusiform | BA 35, BA 36 | -25 | -33 | -20 | 1744 | -24 | -30 | -22 | 0.018 | 4 | Sabsevitz, 2005 (2); Harris, 2006 (1); Hayashi, 2014 (1); Hoffman, 2015 (2) ** |
|  |  |  |  |  |  |  |  |  | -26 | -38 | -16 | 0.017 |  |  |
|  |  |  |  |  |  |  |  |  |  |  |  |  |  |  |
| R | 3 | Parietal | Inferior Parietal  Angular  Precuneus | BA 39 | 46 | -66 | 36 | 1440 | 46 | -68 | 34 | 0.015 | 4 | Sabsevitz, 2005 (2); Rüschemeyer, 2007 (1); van Dam, 2010 (1); Hoffman, 2015 (2) ** |
|  |  |  |  |  |  |  |  |  | 48 | -66 | 44 | 0.012 |  |  |
|  |  |  |  |  |  |  |  |  | 42 | -60 | 36 | 0.012 |  |  |
|  |  |  |  |  |  |  |  |  |  |  |  |  |  |  |
| R | 4 | Limbic | Posterior Cingulate  Parahippocampal | BA 30, BA 29 | 9 | -54 | 10 | 984 | 8 | -54 | 10 | 0.019 | 5 | Sabsevitz, 2005 (1); Harris, 2006 (1); Rüschemeyer, 2007 (1); Rodríguez-Ferreiro, 2011 (1); Hoffman, 2015 (1) ** |
|  |  |  |  |  |  |  |  |  |  |  |  |  |  |  |
| L | 5 | Limbic, Occipital | Posterior Cingulate  Lingual | BA 30, BA 18 | -11 | -56 | 13 | 872 | -12 | -56 | 14 | 0.016 | 4 | Sabsevitz, 2005 (1); Binder, 2005 (1); Harris, 2006 (1); Rüschemeyer, 2007 (1) ** |

**Note:** All the values and labels were extracted from the GingerALE output files. Clusters are ordered for decreasing volume size. Coordinates (x, y, z) are in the MNI space.

**Abbreviations:** H=Hemisphere; ALE = activation likelihood estimation; Nr. = number of studies that contributed to each cluster; L = left; BA = Brodmann area; ** = between brackets are the number of foci from each study that contributed to that specific cluster; R = right

**Fig. 4A** Clusters activated by the **concrete > abstract words -semantic and lexical tasks** – contrast fMRI data.

The crosses are centered in the areas correspond to stereotactic coordinates reported in Table 5.A

The images are presented in neurological convention.

In red the clusters from PET and fMRI data combined, overlaid in green the clusters from fMRI data only.


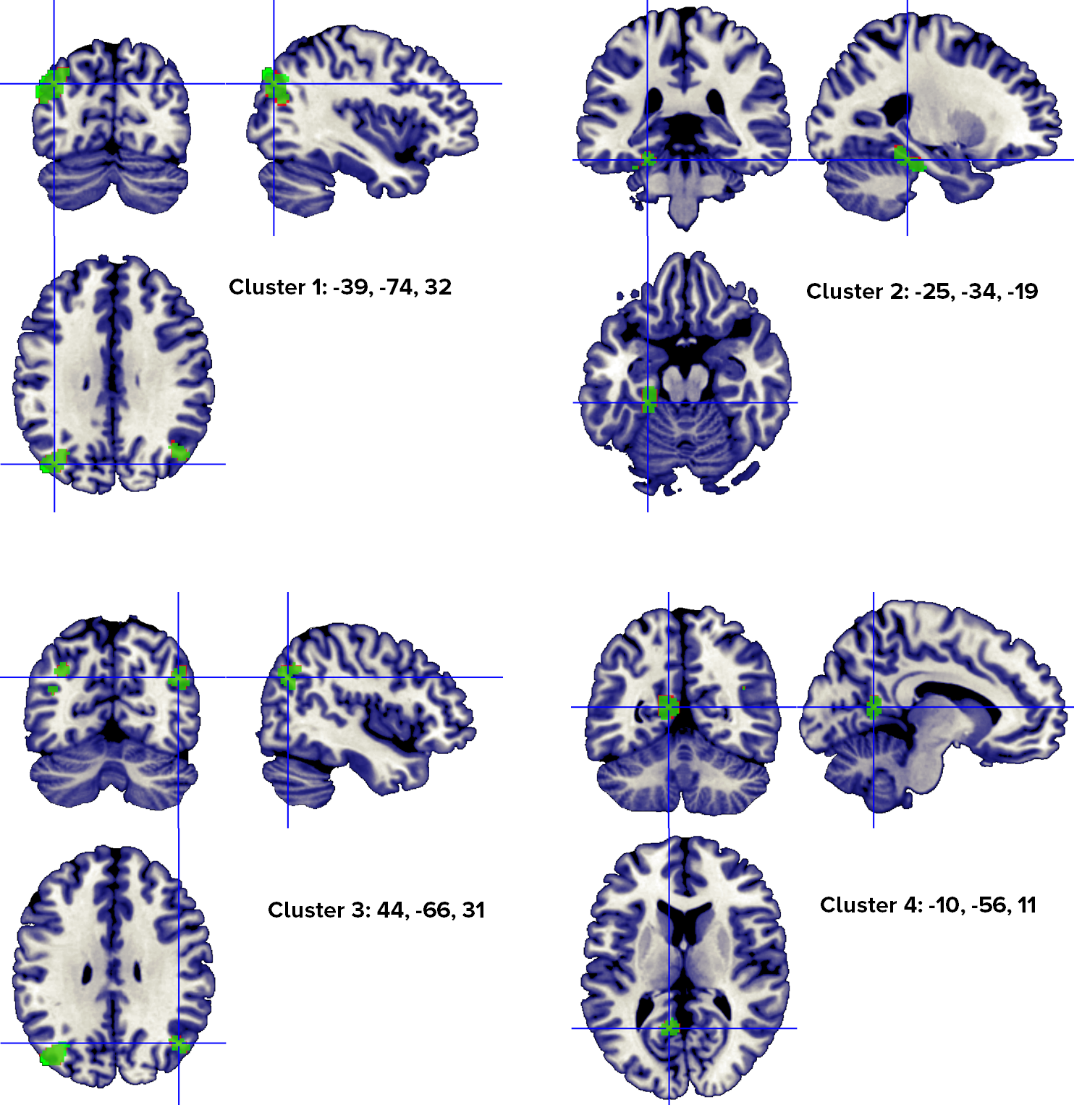


| **Table 5.A \| Concrete > Abstract Words – semantic and lexical tasks only- Clusters, fMRI data**  included 112 stereotactic activation loci from 14 studies, 249 participants  Chosen min. cluster size 656 mm^3^ | | | | | | | | | | | | | | |
| --- | --- | --- | --- | --- | --- | --- | --- | --- | --- | --- | --- | --- | --- | --- |
| **H** | **Cluster** | **Macroanatomical Location** | | **Cytoarchitectonic Label** | **Weighted Center (MNI; mm)** | | | **Vol. (mm3)** | **Peaks: MNI Coordinates (mm)** | | | **ALE score** | **Contributors to cluster** | |
|  |  | **Lobe** | **Gyrus** |  | **x** | **y** | **z** |  | **x** | **y** | **z** |  | **Nr.** | **Studies** |
| L | 1 | Occipital  Temporal,  Parietal | Superior Occipital  Middle Temporal  Precuneus  Angular | BA 19, BA 39 | -39 | -74 | 32 | 4360 | -40 | -74 | 34 | 0.025 | 9 | Sabsevitz, 2005 (2); Binder, 2005 (1); Harris, 2006 (1); van Dam, 2010 (1); Zhuang, 2011 (1); van Dam, 2012 (1); Roxbury, 2014 (1), Skipper, 2014 (5); Hoffman, 2015 (2) ** |
|  |  |  |  |  |  |  |  |  | -46 | -78 | 26 | 0.018 |  |  |
|  |  |  |  |  |  |  |  |  | -40 | -70 | 22 | 0.014 |  |  |
|  |  |  |  |  |  |  |  |  |  |  |  |  |  |  |
| L | 2 | Limbic Lobe  Anterior lobe  Temporal | Parahippocampal  Culmen | BA 35, BA 36 | -25 | -34 | -19 | 1776 | -24 | -38 | -16 | 0.019 | 5 | Sabsevitz, 2005 (2); Harris, 2006 (1); van Dam, 2012 (1); Roxbury, 2014 (1); Hoffman, 2015 (2) ** |
|  |  |  |  |  |  |  |  |  | -24 | -28 | -22 | 0.016 |  |  |
|  |  |  |  |  |  |  |  |  |  |  |  |  |  |  |
| R | 3 | Temporal  Parietal | Middle Temporal  Precuneus | BA 39 | 44 | -66 | 31 | 1680 | 44 | -68 | 32 | 0.017 | 5 | Sabsevitz, 2005 (2); Rüschemeyer, 2007 (1); van Dam, 2010 (1); Roxbury, 2014 (1); Hoffman, 2015 (3) ** |
|  |  |  |  |  |  |  |  |  | 42 | -60 | 36 | 0.012 |  |  |
|  |  |  |  |  |  |  |  |  | 40 | -56 | 24 | 0.009 |  |  |
|  |  |  |  |  |  |  |  |  |  |  |  |  |  |  |
| L | 4 | Limbic Lobe, Occipital | Posterior Cingulate  Lingual | BA 30, BA 18 | -10 | -56 | 11 | 1352 | -10 | -56 | 12 | 0.018 | 5 | Sabsevitz, 2005 (1); Binder, 2005 (2); Harris, 2006 (1); Rüschemeyer, 2007 (1); Roxbury, 2014 (1) ** |
|  |  |  |  |  |  |  |  |  | -8 | -46 | 14 | 0.009 |  |  |

**Note:** All the values and labels were extracted from the GingerALE output files. Clusters are ordered for decreasing volume size. Coordinates (x, y, z) are in the MNI space.

**Abbreviations:** H=Hemisphere; ALE = activation likelihood estimation; Nr. = number of studies that contributed to each cluster; L = left; BA = Brodmann area; ** = between brackets are the number of foci from each study that contributed to that specific cluster; R = right

***ABSTRACT > CONCRETE Meta-analysis***

**Fig. 5.A** Clusters activated by the **abstract > concrete words** contrast, fMRI data.

The crosses are centered in the areas correspond to stereotactic coordinates reported in Table 6.A

The images are presented in neurological convention.

In red the clusters from PET and fMRI data combined, overlaid in green the clusters from fMRI data only.


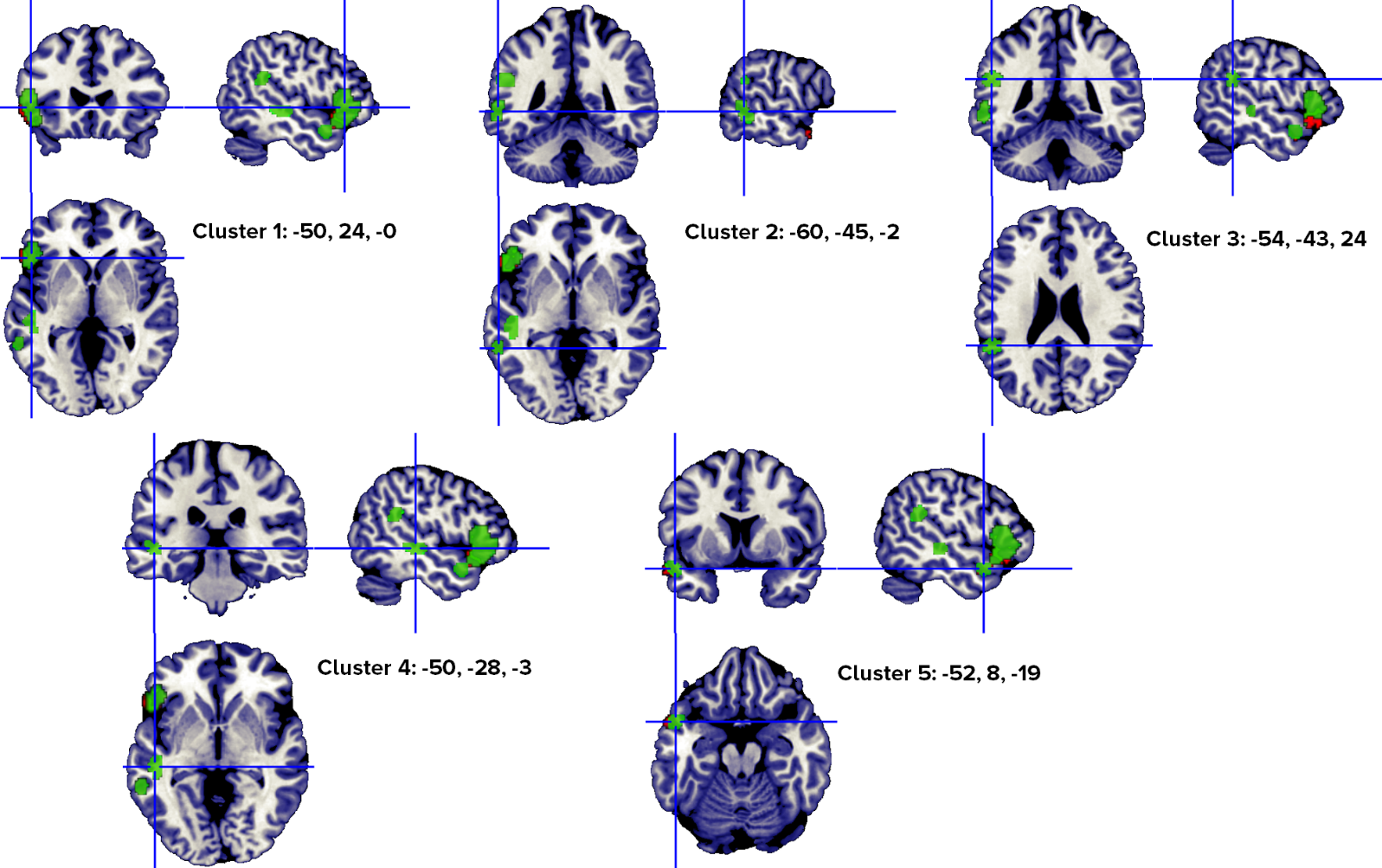


| **Table 6.A\| Abstract > Concrete Word Clusters, fMRI data**  included 133 stereotactic activation loci from 21 studies, 369 participants  Chosen min. cluster size 736 mm^3^ | | | | | | | | | | | | | | |
| --- | --- | --- | --- | --- | --- | --- | --- | --- | --- | --- | --- | --- | --- | --- |
| **H** | **Cluster** | **Macroanatomical Location** | | **Cytoarchitectonic Label** | **Weighted Center (MNI; mm)** | | | **Vol. (mm3)** | **Peaks: MNI Coordinates (mm)** | | | **ALE score** | **Contributors to cluster** | |
|  |  | **Lobe** | **Gyrus** |  | **x** | **y** | **z** |  | **x** | **y** | **z** |  | **Nr.** | **Studies** |
| L | 1 | Frontal,  Temporal | Inferior Frontal  Precentral Gyrus  Superior Temporal | BA 45, BA 47  BA 44 | -50 | 24 | -0.4 | 5136 | -52 | 24 | 4 | 0.030 | 11 | Fiebach, 2004 (1); Sabsevitz, 2005 (3); Binder, 2005 (5); Fliessbach, 2006 (2); Pexman, 2007 (1); Rodríguez-Ferreiro, 2011 (2); Hayashi, 2014 (1); Hoffman, 2015 (3); Skipper, 2014 (2); Wang, 2019 (1); Pauligk, 2019 (2) ** |
|  |  |  |  |  |  |  |  |  | -50 | 22 | -10 | 0.024 |  |  |
|  |  |  |  |  |  |  |  |  |  |  |  |  |  |  |
| L | 2 | Temporal | Middle Temporal | BA 22, BA 21 | -60 | -45 | -2 | 1176 | -60 | -42 | -6 | 0.016 | 5 | Noppeney, 2004 (1); Sabsevitz, 2005 (1); Pexman, 2007 (2); Rodríguez-Ferreiro, 2011 (2); Wang, 2019 (1)** |
|  |  |  |  |  |  |  |  |  | -60 | -48 | 4 | 0.015 |  |  |
|  |  |  |  |  |  |  |  |  |  |  |  |  |  |  |
| L | 3 | Temporal  Parietal | Superior Temporal  Inferior Parietal | BA 13, BA 40 | -54 | -43 | 24 | 1064 | -54 | -42 | 24 | 0.021 | 4 | Hayashi, 2014 (1); Hoffman, 2015 (2); Wang, 2019 (1); Meersmans, 2020 (1)** |
|  |  |  |  |  |  |  |  |  |  |  |  |  |  |  |
| L | 4 | Temporal | Superior and Middle Temporal | BA 22, BA 21 | -50 | -28 | -3 | 904 | -50 | -28 | -4 | 0.016 | 4 | Sabsevitz, 2005 (1); Hoffman, 2015 (2); Kumar, 2016 (1); Wang, 2019 (1)** |
|  |  |  |  |  |  |  |  |  |  |  |  |  |  |  |
| L | 5 | Temporal | Superior and Middle Temporal | BA 38, BA 21 | -52 | 8 | -19 | 784 | -52 | 8 | -20 | 0.015 |  | Sabsevitz, 2005 (1); Binder, 2005 (2); Hoffman, 2015 (1)** |

**Note:** All the values and labels were extracted from the GingerALE output files. Clusters are ordered for decreasing volume size. Coordinates (x, y, z) are in the MNI space.

**Abbreviations:** H=Hemisphere; ALE = activation likelihood estimation; Nr. = number of studies that contributed to each cluster; L = left; BA = Brodmann area; ** = between brackets are the number of foci from each study that contributed to that specific cluster

**Fig. 6.A.** Clusters activated by the **abstract > concrete nouns** contrast.

The crosses are centered in the areas correspond to stereotactic coordinates reported in Table 7.A

The images are presented in neurological convention.

In red the clusters from PET and fMRI data combined, overlaid in green the clusters from fMRI data only.


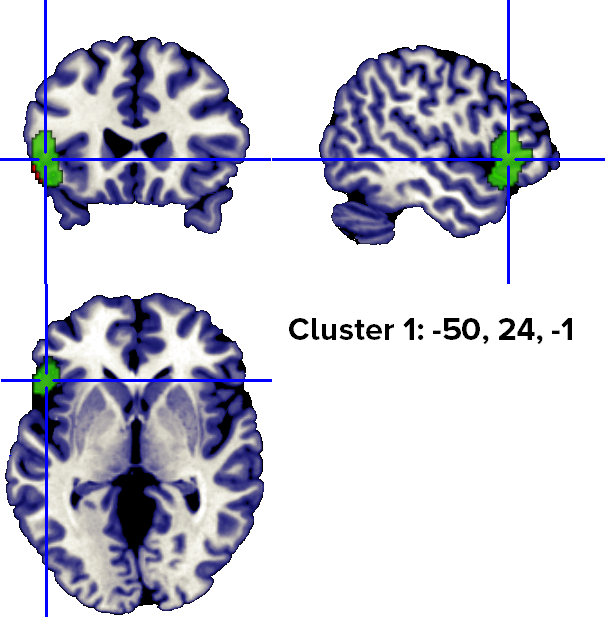


| **Table 7.A \| Abstract > Concrete Nouns Clusters, fMRI data**  included 94 stereotactic activation loci from 16 studies, 301 participants  Chosen min. cluster size 624 mm^3^ | | | | | | | | | | | | | | |
| --- | --- | --- | --- | --- | --- | --- | --- | --- | --- | --- | --- | --- | --- | --- |
| **H** | **Cluster** | **Macroanatomical Location** | | **Cytoarchitectonic Label** | **Weighted Center (MNI; mm)** | | | **Vol. (mm3)** | **Peaks: MNI Coordinates (mm)** | | | **ALE score** | **Contributors to cluster** | |
|  |  | **Lobe** | **Gyrus** |  | **x** | **y** | **z** |  | **x** | **y** | **z** |  | **Nr.** | **Studies** |
| L | 1 | Frontal | Inferior Frontal,  Precentral | BA 47, BA 45, BA 44 | -50 | 24 | -0.9 | 4296 | -52 | 22 | 6 | 0.025 | 9 | Fiebach, 2004 (1); Sabsevitz, 2005 (2); Binder, 2005 (3); Fliessbach, 2006 (2); Pexman, 2007 (1); Hayashi, 2004 (1); Skipper, 2014 (2); Wang, 2019 (1); Pauligk, 2019 (2) ** |
|  |  |  |  |  |  |  |  |  | -48 | 22 | -10 | 0.023 |  |  |

**Note:** All the values and labels were extracted from the GingerALE output files. Clusters are ordered for decreasing volume size. Coordinates (x, y, z) are in the MNI space.

**Abbreviations:** H=Hemisphere; ALE = activation likelihood estimation; Nr. = number of studies that contributed to each cluster; L = left; BA = Brodmann area; ** = between brackets are the number of foci from each study that contributed to that specific cluster

**Fig. 7.A** Clusters activated by the **abstract > concrete words - visual stimuli** – contrast.

The crosses are centered in the areas correspond to stereotactic coordinates reported in Table 8.A

The images are presented in neurological convention.

In red the clusters from PET and fMRI data combined, overlaid in green the clusters from fMRI data only.


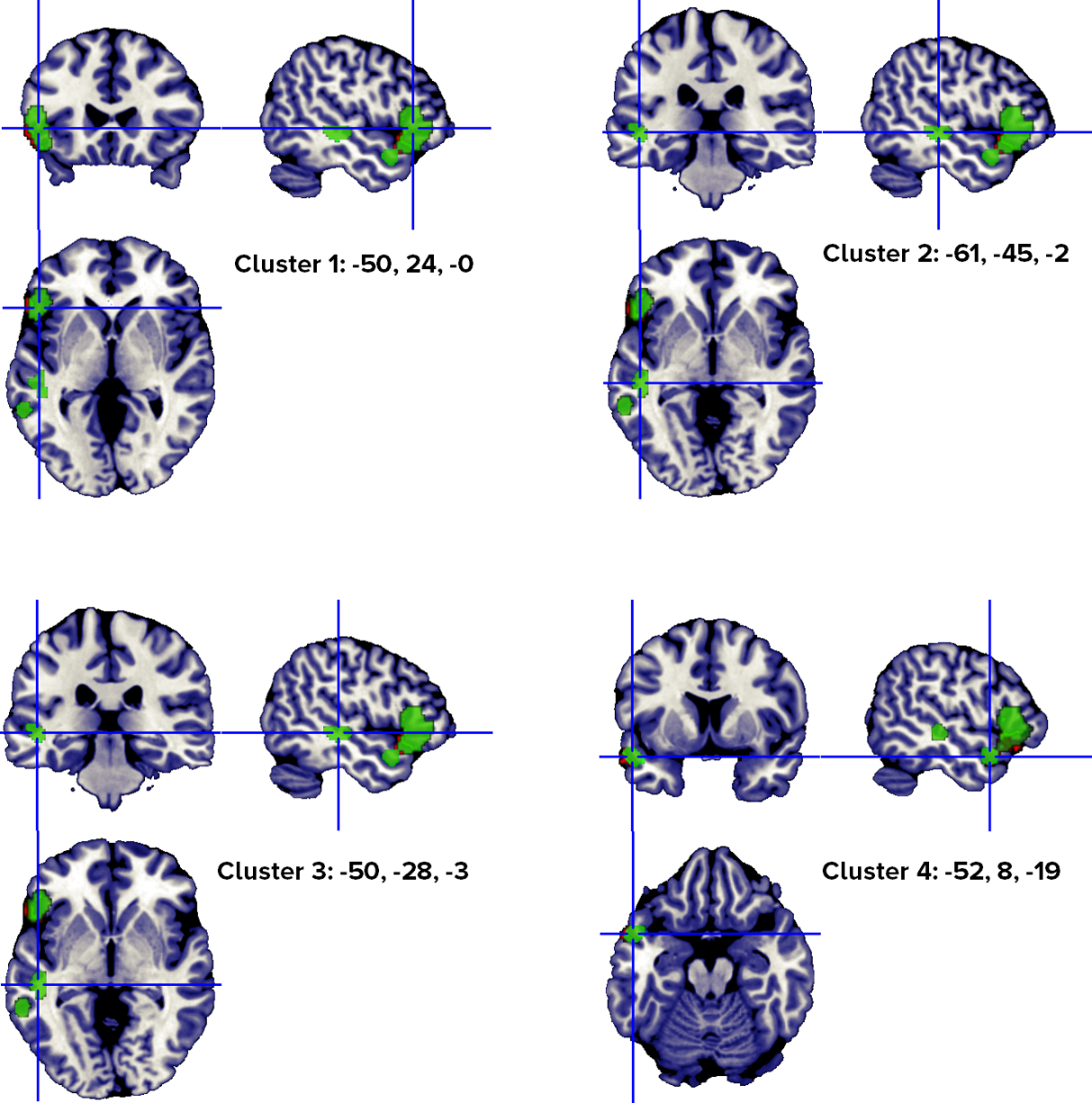


| **Table 8.A \| Abstract > Concrete Words - visual stimuli- Clusters, fMRI data**  included 126 stereotactic activation loci from 19 studies, 336 participants  Chosen min. cluster size 712 mm^3^ | | | | | | | | | | | | | | |
| --- | --- | --- | --- | --- | --- | --- | --- | --- | --- | --- | --- | --- | --- | --- |
| **H** | **Cluster** | **Macroanatomical Location** | | **Cytoarchitectonic Label** | **Weighted Center (MNI; mm)** | | | **Vol. (mm3)** | **Peaks: MNI Coordinates (mm)** | | | **ALE score** | **Contributors to cluster** | |
|  |  | **Lobe** | **Gyrus** |  | **x** | **y** | **z** |  | **x** | **y** | **z** |  | **No.** | **Studies** |
| L | 1 | Frontal,  Temporal | Inferior Frontal  Precentral Gyrus  Superior Temporal | BA 47, BA 45,  BA 44 | -50 | 24 | -0.4 | 5216 | -52 | 24 | 4 | 0.029 | 11 | Fiebach, 2004 (1); Sabsevitz, 2005 (2); Binder, 2005 (5); Fliessbach, 2006 (2); Pexman, 2007 (1); Rodríguez-Ferreiro, 2011 (2); Hayashi, 2014 (1); Hoffman, 2015 (3); Skipper, 2014 (2); Wang, 2019 (1); Pauligk, 2019 (2)** |
|  |  |  |  |  |  |  |  |  | -50 | 22 | -10 | 0.024 |  |  |
|  |  |  |  |  |  |  |  |  |  |  |  |  |  |  |
| L | 2 | Temporal | Middle Temporal | BA 22, BA 21 | -61 | -45 | -2 | 1216 | -60 | -42 | -6 | 0.016 | 5 | Noppeney, 2004 (1); Sabsevitz, 2005 (1); Pexman, 2007 (2); Rodríguez-Ferreiro, 2011 (2); Wang, 2019 (1) ** |
|  |  |  |  |  |  |  |  |  | -60 | -48 | 4 | 0.015 |  |  |
|  |  |  |  |  |  |  |  |  |  |  |  |  |  |  |
| L | 3 | Temporal | Superior Temporal  Middle Temporal | BA 22, BA 21 | -50 | -28 | -3 | 960 | -50 | -28 | -4 | 0.016 | 4 | Sabsevitz, 2005 (1); Hoffman, 2015 (2); Kumar, 2016 (1); Wang, 2019 (1) ** |
|  |  |  |  |  |  |  |  |  |  |  |  |  |  |  |
| L | 4 | Temporal | Superior Temporal  Middle Temporal | BA 38, BA 21 | -53 | 8 | -19 | 840 | -52 | 8 | -20 | 0.015 | 3 | Sabsevitz, 2005 (1); Binder, 2005 (2); Hoffman, 2015 (1) |

**Note:** All the values and labels were extracted from the GingerALE output files. Clusters are ordered for decreasing volume size. Coordinates (x, y, z) are in the MNI space.

**Abbreviations:** H=Hemisphere; ALE = activation likelihood estimation; Nr. = number of studies that contributed to each cluster; L = left; BA = Brodmann area; ** = between brackets are the number of foci from each study that contributed to that specific cluster

**Fig. 8.A** Clusters activated by the **abstract > concrete words -semantic and lexical task**- contrast.

The crosses are centered in the areas correspond to stereotactic coordinates reported in Table 9.A

The images are presented in neurological convention.

In red the clusters from PET and fMRI data combined, overlaid in green the clusters from fMRI data only.

***
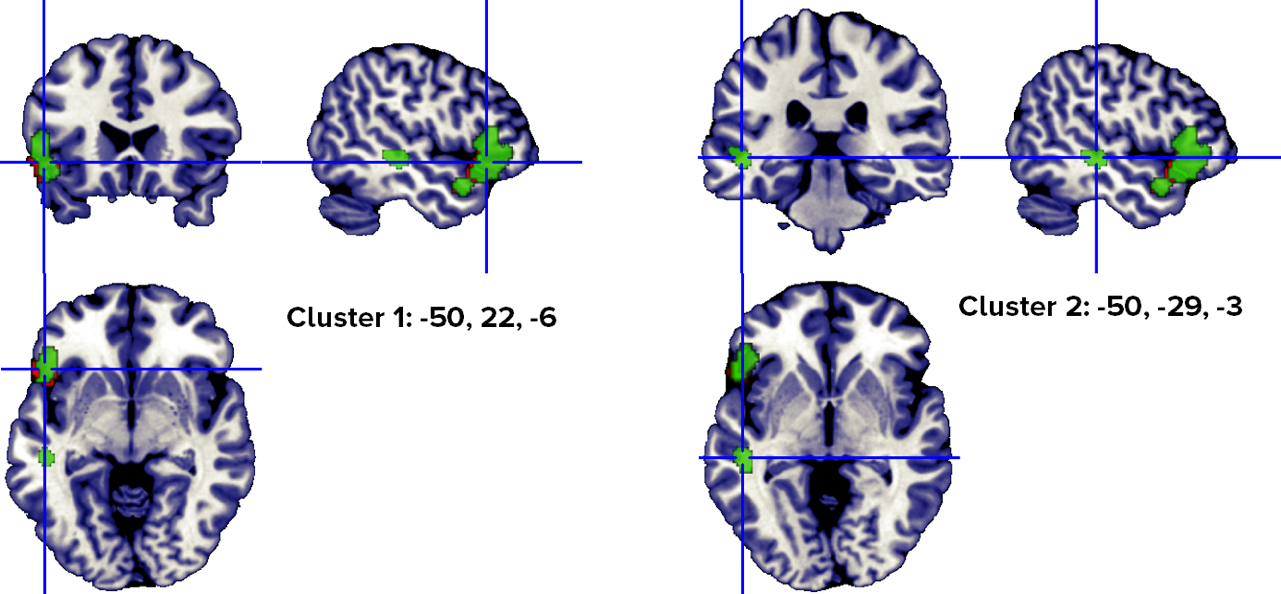
***

| **Table 9.A \| Abstract > Concrete Words – semantic and lexical task only- Clusters, fMRI data**  included 107 stereotactic activation loci from 14 studies, 251 participants  Chosen min. cluster size 640 mm^3^ | | | | | | | | | | | | | | |
| --- | --- | --- | --- | --- | --- | --- | --- | --- | --- | --- | --- | --- | --- | --- |
| **H** | **Cluster** | **Macroanatomical Location** | | **Cytoarchitectonic Label** | **Weighted Center (MNI; mm)** | | | **Vol. (mm3)** | **Peaks: MNI Coordinates (mm)** | | | **ALE score** | **Contributors to cluster** | |
|  |  | **Lobe** | **Gyrus** |  | **x** | **y** | **z** |  | **x** | **y** | **z** |  | **Nr.** | **Studies** |
| L | 1 | Frontal  Temporal | Inferior Frontal,  Superior Temporal | BA 47, BA 45, BA 44 | -50 | 22 | -6 | 5136 | -50 | 22 | -10 | 0.024 | 8 | Fiebach, 2004 (1); Sabsevitz, 2005 (3); Binder, 2005 (4); Pexman, 2007 (1); Hoffman, 2015 (3); Skipper, 2014 (2); Wang, 2019 (1); Pauligk, 2019 (2) ** |
|  |  |  |  |  |  |  |  |  | -48 | 30 | -4 | 0.017 |  |  |
|  |  |  |  |  |  |  |  |  | -52 | 24 | 6 | 0.016 |  |  |
|  |  |  |  |  |  |  |  |  | -52 | 8 | -20 | 0.015 |  |  |
|  |  |  |  |  |  |  |  |  |  |  |  |  |  |  |
| L | 2 | Temporal | Superior Temporal  Middle Temporal | BA 22, BA 21 | -50 | -29 | -3 | 752 | -50 | -28 | -4 | 0.015 | 3 | Sabsevitz, 2005 (1); Hoffman, 2015 (2); Wang, 2019 (1) ** |

**Note:** All the values and labels were extracted from the GingerALE output files. Clusters are ordered for decreasing volume size. Coordinates (x, y, z) are in the MNI space.

**Abbreviations:** H=Hemisphere; ALE = activation likelihood estimation; Nr. = number of studies that contributed to each cluster; L = left; BA = Brodmann area; ** = between brackets are the number of foci from each study that contributed to that specific cluster

| **Table 10.A \| Brodmann areas brief description** | | | |  |
| --- | --- | --- | --- | --- |
| **Cytoarchitectonic Label** | **Constrast** | **Location** | | **Function** |
| **BA** |  |  |  |  |
| BA 13 | A > C | part of the insular cortex (prefrontal lobe) | | plays a coordinating role in interconnecting Wernicke’s and Broca’s areas |
| BA 18 | C > A | secondary visual cortex (occipital lobe) | | processing visual information from v1 |
| BA 19 | C > A | associative visual cortex (occipital lobe) | | feature-extracting, shape recognition, attentional, and multimodal integrating functions |
| BA 20 | A > C | inferior temporal, fusiform and parahippocampal gyri (temporal lobe) | | language understanding and processing: lexico-semantic processing, metaphor comprehension, language comprehension and production, and selective attention to speech |
| BA 21 | A > C | lateral temporal cortex, middle temporal gyrus | | auditory processing and language |
| BA 22 | A > C | posterior superior temporal gyrus, of which the caudal part is usually considered to contain the Wernicke's area | | language processing and primary auditory cortex |
| BA 29 | C > A | retrosplenial cingulate cortex (limbic lobe) | | involved in memory, visuospatial processing, proprioception, and emotion |
| BA 30 | C > A | posterior cingulate & cuneus (limbic lobe) | |  |
| BA 35 | C > A | hippocampal region, perirhinal cortex (rhinal sulcus) | | formation, consolidation, and retrieval of declarative memory |
| BA 36 | C > A | fusiform gyrus, perirhinal cortex (rhinal sulcus) | |  |
| BA 38 | A > C | rostral part of the superior and middle temporal gyri (temporal pole) | | semantic processing, speech comprehension, and naming |
| BA 39 | C > A | angular gyrus, considered by some to be part of Wernicke's area (inferior parietal lobe) | | reading-related tasks, writing, seems to also participate in an executive function brain circuitry |
| BA 40 | A > C | supramarginal gyrus considered by some to be part of Wernicke's area posterior parietal cortex | | reading-related tasks, language comprehension |
| BA 44 | A > C | pars opercularis, part of the inferior frontal gyrus and part of Broca's area (frontal cortex) | | verbal fluency, phonological processing, grammar processing, attention in speech, sentence comprehension |
| BA 45 | A > C | pars triangularis, part of the inferior frontal gyrus and part of Broca's area (frontal cortex) | | motor speech programming, semantic decision tasks |
| BA 47 | A > C | pars orbitalis - inferior frontal gyrus | | semantic processing, phonological processing, semantic encoding |

**Abbreviations:** BA = Brodmann area; C > A = concrete > abstract contrasts; A > C = abstract > concrete contrasts

**Figure legends:**

For anatomical labeling and figures, we capitalized on the Automatic Anatomical Labeling (AAL) template available in the MRIcron visualization Software - Windows 64 bit (v1.0.20190902) (https://www.nitrc.org/projects/mricron).

Fig. 1.A Clusters activated by the concrete > abstract words contrast, fMRI data. The crosses are centered in the areas correspond to stereotactic coordinates reported in Table 2. The images are presented in neurological convention.

Fig. 2.A Clusters activated by the concrete > abstract nouns contrast, fMRI data. The crosses are centered in the areas correspond to stereotactic coordinates reported in Table 3. The images are presented in neurological convention.

Fig. 3.A Clusters activated by the concrete > abstract words - visual stimuli – contrast, fMRI data. The crosses are centered in the areas correspond to stereotactic coordinates reported in Table 4. The images are presented in neurological convention.

Fig. 4.A Clusters activated by the concrete > abstract words -semantic and lexical tasks – contrast, fMRI data. The crosses are centered in the areas correspond to stereotactic coordinates reported in Table 5. The images are presented in neurological convention.

Fig. 5.A Clusters activated by the abstract > concrete words contrast, fMRI data. The crosses are centered in the areas correspond to stereotactic coordinates reported in Table 6. The images are presented in neurological convention.

Fig. 6.A Clusters activated by the abstract > concrete nouns contrast, fMRI data. The crosses are centered in the areas correspond to stereotactic coordinates reported in Table 7. The images are presented in neurological convention.

Fig. 7.A Clusters activated by the abstract > concrete words - visual stimuli – contrast, fMRI data. The crosses are centered in the areas correspond to stereotactic coordinates reported in Table 8. The images are presented in neurological convention.

Fig. 8.A Clusters activated by the abstract > concrete words -semantic and lexical task- contrast, fMRI data. The crosses are centered in the areas correspond to stereotactic coordinates reported in Table 9. The images are presented in neurological convention.

**Tables legends:**

Table 1.A | Descriptive information of the 28 experiments included in the meta-analysis

Table 2.A |Concrete > Abstract Word Clusters, fMRI data

Table 3.A |Concrete > Abstract Nouns Clusters, fMRI data

Table 4.A |Concrete > Abstract Words - Visual stimuli- Clusters, fMRI data

Table 5.A | Concrete > Abstract Words – semantic and lexical tasks only- Clusters, fMRI data

Table 6.A | Abstract > Concrete Word Clusters, fMRI data

Table 7.A | Abstract > Concrete Nouns Clusters, fMRI data

Table 8.A | Abstract > Concrete Words - visual stimuli- Clusters, fMRI data

Table 9.A | Abstract > Concrete Words – semantic and lexical task only- Clusters, fMRI data

Table 10.A | Brodmann areas brief description
